# Visualization of oxidized guanine nucleotides accumulation in living cells with split MutT

**DOI:** 10.1093/nar/gkae371

**Published:** 2024-05-13

**Authors:** Yoshihiro Fujikawa, Hidehiko Kawai, Tetsuya Suzuki, Hiroyuki Kamiya

**Affiliations:** Graduate School of Biomedical and Health Sciences, Hiroshima University, 1-2-3 Kasumi, Minami-ku, Hiroshima 734-8553, Japan; Graduate School of Biomedical and Health Sciences, Hiroshima University, 1-2-3 Kasumi, Minami-ku, Hiroshima 734-8553, Japan; Graduate School of Biomedical and Health Sciences, Hiroshima University, 1-2-3 Kasumi, Minami-ku, Hiroshima 734-8553, Japan; Graduate School of Biomedical and Health Sciences, Hiroshima University, 1-2-3 Kasumi, Minami-ku, Hiroshima 734-8553, Japan

## Abstract

Cancer cells produce vast quantities of reactive oxygen species, leading to the accumulation of toxic nucleotides as 8-oxo-7,8-dihydro-2′-deoxyguanosine 5′-triphosphate (8-oxo-dGTP). The human MTH1 protein catalyzes the hydrolysis of 8-oxo-dGTP, and cancer cells are dependent on MTH1 for their survival. MTH1 inhibitors are possible candidates for a class of anticancer drugs; however, a reliable screening system using live cells has not been developed. Here we report a visualization method for 8-oxo-dGTP and its related nucleotides in living cells. *Escherichia coli* MutT, a functional homologue of MTH1, is divided into the N-terminal (1–95) and C-terminal (96–129) parts (Mu95 and 96tT, respectively). Mu95 and 96tT were fused to Ash (assembly helper tag) and hAG (Azami Green), respectively, to visualize the nucleotides as fluorescent foci formed upon the Ash-hAG association. The foci were highly increased when human cells expressing Ash-Mu95 and hAG-96tT were treated with 8-oxo-7,8-dihydro-2′-deoxyguanosine (8-oxo-dG) and 8-oxo-dGTP. The foci formation by 8-oxo-dG(TP) was strikingly enhanced by the MTH1 knockdown. Moreover, known MTH1 inhibitors and oxidizing reagents also increased foci. This is the first system that visualizes damaged nucleotides in living cells, provides an excellent detection method for the oxidized nucleotides and oxidative stress, and enables high throughput screening for MTH1 inhibitors.

## Graphical Abstract

**Graphical Abstract:**
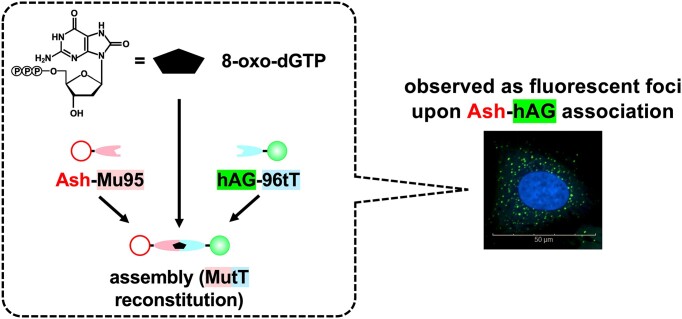

## Introduction

Mutations are caused by various factors, including nucleobase damage in DNA and its precursors (1,2). An oxidatively damaged base, 8-oxo-7,8-dihydroguanine (8-oxo-Gua, also known as 8-hydroxyguanine), is one of the major base lesions produced by reactive oxygen species (ROS) (3). In mammalian cells, this oxidized base causes base substitution mutations including G:C→T:A transversion mutations at the modified sites, and untargeted base substitution mutations at G:C sites in DNA (4–9). The oxidation of dGTP produces 8-oxo-7,8-dihydro-2′-deoxyguanosine 5′-triphosphate (8-oxo-dGTP). When DNA polymerases incorporate 8-oxo-dGTP, A:T→C:G transversion mutations are induced (10). The *Escherichia coli* MutT and mammalian MTH1 (NUDT1) proteins catalyze the hydrolysis of 8-oxo-dGTP (11,12), and the deficiencies of MutT and MTH1 cause elevated incidence of spontaneous mutations and tumors, respectively (13,14).

Oncogene activation leads to ROS production, and consequently cancer cells accumulate toxic nucleotides including 8-oxo-dGTP (15–18). MTH1 is highly important for cancer cell survival because of its hydrolytic activity for 8-oxo-dGTP. Thus, MTH1 inhibitors are expected to be a class of anticancer drug candidates (19,20). Meanwhile, human cells possess at least two additional hydrolyzing enzymes specific for 8-oxo-7,8-dihydro-2′-deoxyguanosine (8-oxo-dG) nucleotides: MTH2 (NUDT15) and NUDT5 (21–23). The substrates for MTH1 are 8-oxo-dGTP, 2-oxo-1,2-dihydro-2′-deoxyadenosine 5′-triphosphate, and 8-oxo-7,8-dihydro-2′-deoxyadenosine 5′-triphosphate (24). MTH2 catalyzes the hydrolysis of 8-oxo-dGTP (21). NUDT5 is a nucleoside diphosphate phosphatase and its major substrates are 8-oxo-7,8-dihydro-2′-deoxyguanosine 5′-diphosphate (8-oxo-dGDP) and 8-oxo-7,8-dihydro-2′-deoxyadenosine 5′-diphosphate (22,25). The importance of the NUDT5 8-oxo-dGDPase activity is indicated by (i) the decreased spontaneous mutation frequency in *mutT*-deficient *E. coli* cells by the expression of NUDT5 and (ii) the increased mutant frequency induced by exogenous 8-oxo-dGTP in NUDT5-knockdown human cells (22,23). The common substrates for these proteins are 8-oxo-dG nucleotides and thus, a high-throughput screening system using live cells containing these proteins is required to identify effective lead compounds. However, a reliable screening system using live cells has been absent. Moreover, ROS also oxidize ribonucleotides (RNA precursors) that possibly induce transcription errors (26). The oxidized ribonucleotides are substrates for MTH1 and NUDT5 (27,28) and seem responsible for the MTH1-dependency of cancer cells at least in part.

The *E. coli* MutT protein is a hydrolyzing enzyme for 8-oxo-dGTP as described above. Its *K*_m_ value is 0.081 μM, much lower than that of MTH1 (∼10 μM) (24,29). In addition, 8-oxo-dGDP, 8-oxo-7,8-dihydroguanosine 5′-triphosphate (8-oxo-rGTP) and 8-oxo-7,8-dihydroguanosine 5′-diphosphate (8-oxo-rGDP) are also substrates for MutT (29). The *K*_m_ values are 0.058, 0.26 and 0.045 μM, respectively. Thus, MutT seems to bind these oxidized nucleotides with high affinities.

**Figure 1.**
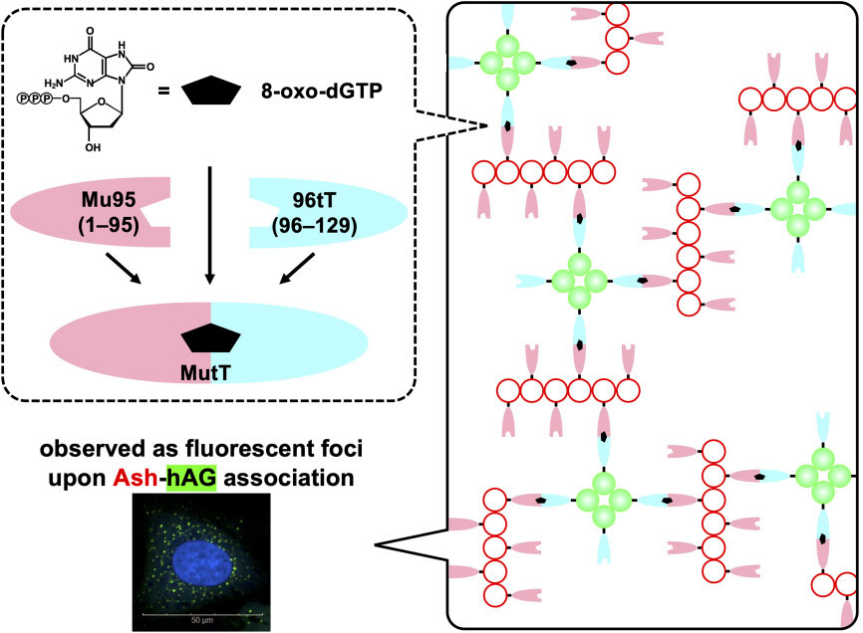
Conceptual illustration of 8-oxo-dGTP-induced fluorescent foci formation. The nucleotide induces Mu95 (pink semi-ellipse) and 96tT (pale blue semi-ellipse) association. The Ash (white circle) and hAG (green circle) proteins are fused to Mu95 and 96tT, respectively, and the formation of the ternary complex promotes the Ash-hAG crosslinking. Due to the self-association properties of Ash and hAG, the crosslinking induces a fluorescent focus.

In this study, we developed a visualization method for the accumulation of 8-oxo-dGTP and the related compounds in living cells. We divided the *E. coli* MutT protein into the N-terminal (1–95) and C-terminal (96–129) parts (Mu95 and 96tT, respectively) based on the ternary structure (split MutT) (30,31). The Glu-53 residue was replaced by Ala to inactivate the catalytic function (31–33). Ash (assembly helper tag) and hAG (Azami Green) are fused to Mu95 and 96tT, respectively. The hAG protein is a tetrameric fluorescent protein and the Ash protein forms high-molecular-weight homo-oligomers (34). The Fluoppi method uses Ash-X/X-Ash and hAG-Y/Y-hAG fusion proteins. The two fusion proteins build crosslinks when X and Y interact, resulting in the fluorescent foci. We hypothesized that the assembly of Mu95 and 96tT is promoted in the presence of 8-oxo-dGTP, and consequently the association of Ash and hAG is increased (Figure 1). Indeed, the foci formation was highly promoted when human U2OS cells expressing Ash-Mu95 and hAG-96tT were treated with 8-oxo-dG and 8-oxo-dGTP and MTH1 knockdown further enhanced the foci formation. Moreover, known MTH1 inhibitors and oxidizing reagents also increased foci. The split MutT protein-expressing cells would be useful for the visualization of 8-oxo-dGTP and the related compounds and screening of anticancer drug candidates. Moreover, to our knowledge, this system is the first one that detects/visualizes damaged nucleotides in living cells.

## Materials and methods

### Linker mutagenesis and 8-oxo-dGTPase assay

The construction of the gene for MutT containing a Gly-Ser linker will be reported elsewhere. The mutant gene has the 5′-ATGCAGGGTGGAGGCGGTTCAGGCGGTGGAGGCTCCGGAGGTGGCGGAAGTGGCGGTGGCGGATCAGGTGGAGGTGGCAGCGGAGGCGGAGGTTCCGGATCT-3′ sequence encoding the Met-Gln-(Gly-Gly-Gly-Gly-Ser)_6_-Gly-Ser linker between codons 95 and 96 (Supplementary Figure S1). The wild-type and mutant *mutT* genes were inserted into pET16b (Merck, Darmstadt, Germany) using the *Nde*I and *Bam*HI sites. *E. coli* BL21(DE3) cells transformed by the *mutT* gene were cultured in LB medium containing ampicillin (50 μg/ml) and isopropyl-β-d-thiogalactopyranoside (1 mM) at 37°C for 4 h. The bacterial cells were treated with lysozyme and DNase I. The amino-terminal His-tagged MutT protein in the insoluble fraction was dissolved in SDS-Lysis buffer (50 mM Tris–HCl (pH 8.0), 100 mM NaCl, 5 mM MgCl_2_, 2% SDS) and renatured by removing SDS, according to the report by He and Ohnishi (35). The protein was purified by the Capturem His-Tagged Purification Maxiprep kit (Takara Bio, Kusatsu, Japan).

The 8-oxo-dGTPase activity was measured in a reaction mixture (100 μl) containing 20 mM Tris–HCl (pH 7.5), 8 mM MgCl_2_, 5 mM DTT, 4% glycerol, 80 μg/ml bovine serum albumin, 10 μM 8-oxo-dGTP, and 1.5 nM MutT. The mixtures were incubated at 30°C for 10 min and the reactions were terminated by the addition of 100 μl of 0.5% SDS. The ratios of 8-oxo-7,8-dihydro-2′-deoxyguanosine 5′-monophosphate (8-oxo-dGMP) and 8-oxo-dGTP were analyzed by anion exchange chromatography as described previously (24).

### Cell culture

The human osteosarcoma U2OS cells (ATCC HTB-96) were obtained from the American Type Culture Collection (Manassas, Virginia, USA). U2OS cells were cultured in Dulbecco's modified Eagle's medium (DMEM, Nissui Pharmaceutical, Tokyo, Japan), supplemented with 10% (v/v) fetal bovine serum (bioSera, Cholet, France), 0.523 g/l l-glutamine and 1.44 g/l NaHCO_3_ under a 5% CO_2_ atmosphere at 37°C. The medium described above was routinely used unless stated otherwise.

### Fluorescent foci quantitation

Fluorescent images of 4% paraformaldehyde-fixed cells plated on 96-well imaging plates (CellCarrier-96, PerkinElmer, Waltham, Massachusetts, USA) were acquired by an Opera Phenix High Content Screening System (PerkinElmer), using 20× and 63× water immersion objective lenses. Hoechst 33342 and hAG were excited by 405- and 488-nm lasers and fluorescence was detected within the wavelength ranges of 435–480 and 500–540 nm, respectively. Confocal Z-stack images of five planes with 1.5-μm distances were acquired for 25–49 fields per well. The fluorescence images of cells were extracted with the same contrast settings during the same experiment. The fluorescence images were analyzed by the Harmony Analysis Software version 4.9 (PerkinElmer). The object parameters of the individual nuclei, such as roundness, length, intensity, and area, were compared among all nuclei in the analysis. Only cells with a standard nucleus were used for the subsequent analysis. In the transient expression experiment, only cells with hAG expression were gated and used for the subsequent analysis. The individual cell area was detected by the software, and the number of foci composed of Ash-hAG in the area was graphically and statistically analyzed by the Spotfire Software version 10.10.3 (TIBCO, Palo Alto, California, USA) and BellCurve for Excel version 4.05 (Social Survey Research Information, Tokyo, Japan).

### Plasmid DNAs for transient expression

The genes for Mu95 and 96tT were amplified by PCR, using the ‘codon-humanized’ gene for MutT (Ala53) (Integrated DNA Technologies, Coralville, Iowa, USA, Supplementary Figure S2) as the template and the primers listed in Supplementary Table S1. The PCR products were inserted into the vector plasmid DNAs, pAsh-MCL, pAsh-MNL ver.2, phAG-MCL and phAG-MNL (Medical & Biological Laboratories, Tokyo, Japan, Fluoppi ver. 2), using the *Pst*I and *Xho*I sites. Plasmid DNAs with the correct sequences were isolated from *E. coli* DH5α (Takara Bio) and purified with a Qiagen Plasmid Midiprep kit (Qiagen, Hilden, Germany), using the protocol for endotoxin-free DNA. The constructed plasmid DNAs are shown in Supplementary Table S2.

### Split MutT (sMutT) cells

**Figure 2.**
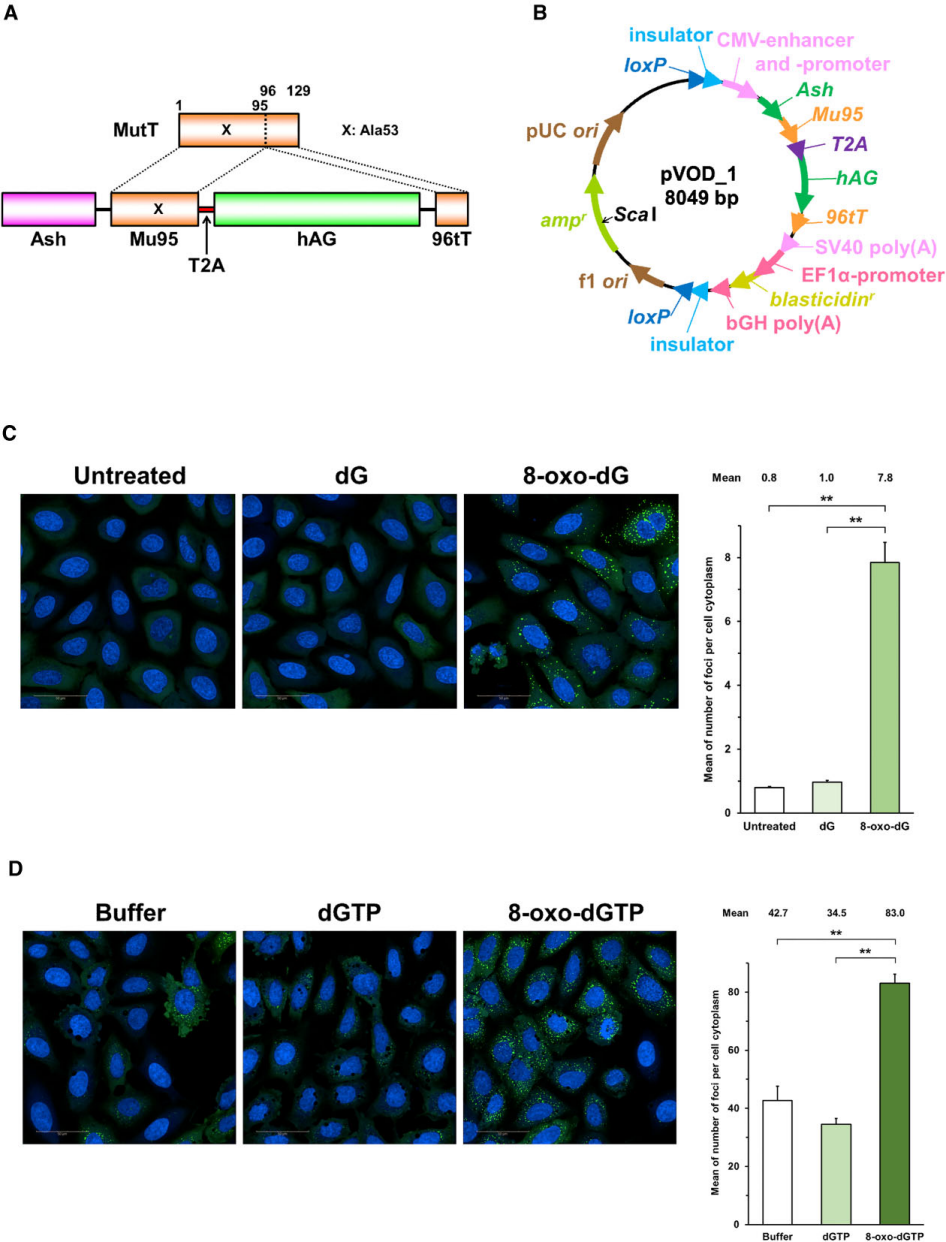
Fluorescent foci formation in sMutT cells expressing the split MutT. (**A**) Structure of the split *mutT* gene. (**B**) The pVOD_1 plasmid used for sMutT cell preparation. *amp^r^*, ampicillin-resistance gene; *blasticidin^r^*, blasticidin S-resistance gene. The *Sca*I site used for linearization is present in the *amp^r^* gene. (**C**) Foci formation by 8-oxo-dG nucleoside treatment. (**D**) Foci formation by 8-oxo-dGTP introduction. The nuclei were stained by Hoechst 33342. The white bars in the images indicate 50 μm. Each mean values of the number of foci per cell cytoplasm obtained in three independent experiments shown in (**C**) Supplementary Figure S5A and (**D**) Supplementary Figure S5B were analyzed as single data. Data are represented as the means with standard deviations. **P< 0.01 (Turkey–Kramer test).

The pLSODN-4D-MCS1-Blast-MCS2 plasmid was constructed by assembling MCS1-EF1α-Blasticidin-pA-MCS2-PGK-hsvTK (System Biosciences, Palo Alto, California, USA) and pLSODN-4D (BioDynamics Laboratory, Tokyo, Japan, Long ssDNA Preparation kit for 3.0 kb) and will be reported elsewhere. The pVOD_1 plasmid including the *Ash-Mu95*-*T2A*-*hAG-96tT* gene (Figure 2A and B, Supplementary Table S2, and Supplementary Figure S4) was constructed by assembling PCR fragments using an NEBuilder HiFi DNA Assembly Cloning kit (New England Biolabs, Ipswich, Massachusetts, USA), according to the manufacturer's protocol. The PCR primers used for plasmid construction are listed in Supplementary Table S1. The templates were pAsh-Mu95HC (for CMV enhancer-promoter-*Ash-Mu95HC*), PB-CMV-MCS-EF1α-GreenPuro cDNA cloning and expression vector (System Biosciences, for *T2A*), phAG-96tTHC (for *hAG-96tTHC*-poly(A) signal), and pLSODN-4D-MCS1-Blast-MCS2 (for *loxP*-insulator, EF1α promoter-blasticidin S resistance gene-insulator-*loxP*, and f1 *ori*-ampicillin resistance gene-pUC *ori*). The desired DNA fragments were amplified using KOD One PCR Master Mix (Toyobo, Osaka, Japan). The constructed plasmid with the correct sequence was isolated from *E. coli* HST08 (Takara Bio) and purified with a GenElute HP Plasmid Miniprep kit (Merck).

To establish sMutT cells that stably express Ash-Mu95-T2A-hAG-96tT, the constructed plasmid was linearized by *Sca*I digestion (Figure 2B) and transfected into U2OS cells with Lipofectamine 3000 (Thermo Fisher Scientific, Waltham, Massachusetts, USA), according to the manufacturer's instructions. Cells with the *Ash-Mu95*-*T2A*-*hAG-96tT* gene integrated in the genome were selected using 5 μg/ml blasticidin (InvivoGen, San Diego, California, USA) for the isolation of single cell clones.

### 8-Oxo-dG treatment (transient expression of Mu95- and 96tT-fusion proteins)

U2OS cells (1.0 × 10^4^ cells) were plated on 96-well imaging plates and cultured overnight in minimum essential medium Eagle, α modification (α-MEM, Merck) supplemented with 10% (v/v) fetal bovine serum (Nichirei Bioscience, Tokyo, Japan) and 0.292 g/l L-glutamine. The Mu95 and 96tT plasmids were transfected by Lipofectamine 3000 according to the manufacturer's instructions, and the cells were incubated at 37°C under a 5% CO_2_ atmosphere for 5 h. After medium exchange, the cells were cultured for an additional 18.5 h. The 8-oxo-dG nucleoside (final concentrations of 1–100 μM, Fujifilm-Wako, Osaka, Japan) was added to the medium, and the cells were cultured for 48 h. The cells were fixed with 4% paraformaldehyde phosphate buffer solution (Nacalai Tesque, Kyoto, Japan) at 4°C overnight, and the nuclei were stained with 1 μg/ml Hoechst 33342 (Thermo Fisher Scientific)–0.05% Triton X-100 (Nacalai Tesque). Fluorescent foci quantitation was conducted as described above.

### 8-Oxo-dG and 8-oxo-dGTP treatments (sMutT cells)

For the 8-oxo-dG treatment, sMutT cells (1.0 × 10^4^ cells) were plated on 96-well imaging plates for 48 h and dG (Fujifilm-Wako) or 8-oxo-dG was then added to the medium (final concentration of 50 μM). The cells were cultured for 30 min at 37°C under a 5% CO_2_ atmosphere, and fixed with 4% paraformaldehyde at 4°C overnight. The nuclei were stained by Hoechst 33342–Triton X-100. Fluorescent foci quantitation was conducted as described above.

The 8-oxo-dGTP introduction was performed according to the methods described previously (23,36). sMutT cells (1.0 × 10^4^ cells) were cultured for 48 h and treated with KH buffer containing 2 mM dGTP (Cytiva, Marlborough, Massachusetts, USA) or 8-oxo-dGTP (TriLink Biotechnologies, San Diego, California, USA), at 37°C for 15 min under a 5% CO_2_ atmosphere. After the medium was exchanged, the cells were incubated at 37°C for 15 min. The fixation and nuclei staining were then conducted as described above.

### Live-cell imaging

sMutT cells (1.0 × 10^6^ cells) were cultured in α-MEM overnight in 35-mm glass bottom dishes (AGC Techno Glass, Yoshida-Cho, Japan, Iwaki 3911–035). One hour before imaging, the medium was replaced by fresh α-MEM. Live-cell images were obtained by using an Olympus LCV110 microscope system (40 × objective lens) equipped with an incubator (37°C under a 5% CO_2_ atmosphere). The dishes were set up in a tray for the LCV110 system and preincubated for 1 h. Images were acquired at intervals of 4–5 min between each acquisition. Either 4× concentrated dG or 8-oxo-dG was added to the medium in the dish (final concentration 100 μM) at 30 min after the start of recording, and the images were acquired continuously at the indicated intervals for 3 h (total 3.5 h). The foci composed of Ash-hAG were excited by a 470–490-nm laser, and fluorescence was detected within the 515–550 nm wavelength range. Z-stack images of three planes with 1.0-μm distances were acquired. Images were processed and assembled into movies with the MetaMorph software version 7.7.5 (Molecular Devices, San Jose, California, USA).

### Knockdown of MTH1/MTH2/NUDT5 and western blotting

sMutT cells (1.0 × 10^5^ cells) were reverse transfected in a 12-well plate with 5 nM siRNA complexed with Lipofectamine RNAiMAX (Thermo Fisher Scientific). The siRNAs against MTH1, MTH2 and NUDT5 (23) (6 pmol, Thermo Fisher Scientific, Supplementary Table S3) and Lipofectamine RNAiMAX (0.6 μl) in Opti-MEM I Reduced Serum Medium (total volume 200 μl, Thermo Fisher Scientific) were mixed, left for 5 min, and added to the cells (volume 1 ml) immediately after they were plated. Stealth RNAi siRNA Negative Control, Med GC (Thermo Fisher Scientific) was used as the control RNA. At 24 h after reverse transfection, the complex was removed, the medium was changed to fresh DMEM, and the cells were cultured at 37°C for 24 or 28 h.

For western blotting, cells were harvested by trypsinization at 24, 48 and 52 h after siRNA reverse transfection. Cells were lysed by radioimmunoprecipitation (RIPA) buffer to obtain whole cell extracts. The extracts were fractionated by 0.1% SDS–10% (NUDT5) or 12.5% (MTH1 and MTH2) polyacrylamide gel electrophoresis, and proteins were transferred to PVDF membranes (Merck). The membranes were then blocked with 5% skimmed milk in phosphate-buffered saline containing 0.05% Tween 20 (PBS-T), for 1 h at room temperature. The mouse anti-MTH1, mouse anti-NUDT5, rabbit anti-MTH2, and mouse anti-β-tubulin antibodies were purchased from Santa Cruz Biotechnology (Santa Cruz, California, USA, catalog nos. sc-271082 and sc-398644), Proteintech (Rosemont, Illinois, USA, catalog no. 25914-1-AP), and Fujifilm Wako (catalog no. 014-25041), respectively, and used as the primary antibodies. Anti-mouse and anti-rabbit IgGs conjugated with horseradish peroxidase (Cytiva, catalog nos. NA931 and NA934) were used as the secondary antibodies. The final concentrations of the anti-MTH1, anti-NUDT5, anti-MTH2, and anti-β-tubulin antibodies were 2, 2, 0.25 and 100 ng/μl, respectively. The anti-IgG antibody was added to a dilution of 1:5000 in its working solution. The proteins were detected with the ImmunoStar LD reagent (Fujifilm Wako) for MTH1, MTH2 and NUDT5, and the Chemi-Lumi One Super reagent (Nacalai Tesque) for β-tubulin, scanned with an ImageQuant LAS 4000 mini image analyzer (GE Healthcare, Piscataway, New Jersey, USA), and quantified with the ImageJ software (37).

### Knockdown of MTH1/MTH2/NUDT5 and 8-oxo-dG treatment

For fluorescent foci quantitation, sMutT cells (1.0 × 10^4^ cells) in 96-well imaging plates were reverse transfected with 5 nM siRNAs complexed with Lipofectamine RNAiMAX. The siRNAs against MTH1, MTH2, and NUDT5 (5 pmol) and Lipofectamine RNAiMAX (0.05 μl) in Opti-MEM I Reduced Serum Medium (total volume of 30 μl) were mixed, left for 5 min, and added to the cells (70 μl) immediately after they were plated. Stealth RNAi siRNA Negative Control, Med GC was used as the control RNA. At 24 h after reverse transfection, the complex was removed, the medium was changed to 100 μl DMEM, and the cells were cultured at 37°C for 24 h. The medium was replaced by fresh DMEM before dG or 8-oxo-dG addition. Either 2′-deoxynucleoside (final concentration of 100 μM) was added to the medium, and the cells were incubated at 37°C for 30 m under a 5% CO_2_ atmosphere. For quantitation, the cells were fixed with 4% paraformaldehyde at 4°C overnight, and the nuclei were stained by Hoechst 33342-Triton X-100. Fluorescent foci quantitation was conducted as described above.

For live-cell imaging, siRNA knockdown experiments in a 12-well plate were conducted as described above. At 24 h after reverse transfection, cells were harvested by trypsinization and resuspended in α-MEM. The cells (3.5 × 10^4^ cells) were re-plated in a droplet placed on the center of a 35-mm glass bottom dish and cultured for 24 h. Live-cell imaging with dG or 8-oxo-dG treatment was conducted as described above.

### Knockdown of MTH1 and 8-oxo-dGTP treatment

The siRNA knockdown experiments in the 96-well imaging plate and the 8-oxo-dGTP treatments were conducted as described above.

### Treatment with oxidizing reagents

sMutT cells (5.0 × 10^3^ cells) were cultured overnight in 96-well imaging plates and then in serum-free DMEM for 5 days. The medium was exchanged to pyruvate-free DMEM (Merck) supplemented with 0.584 g/l l-glutamine, before the addition of oxidizing reagents. The cells were treated with 3 mM KBrO_3_ (Merck) or 30 μM H_2_O_2_ (Fujifilm-Wako) for 6 h. Afterwards, the cells were fixed with 4% paraformaldehyde at 4°C overnight, and the nuclei were stained by Hoechst 33342–Triton X-100. Fluorescent foci quantitation was conducted as described above.

### Treatment with MTH1 inhibitors

sMutT cells (5.0 × 10^3^ cells) were cultured overnight in 96-well imaging plates and then in serum-free DMEM for 5 days. TH287 (Selleck, Houston, Texas, USA) and (*S*)-crizotinib (Selleck) were added to the medium (final concentrations 100 μM and 5 μM, respectively) and the cells were incubated for 6 h. The cells were treated with 4% paraformaldehyde at 4°C overnight, and the nuclei were stained by Hoechst 33342–Triton X-100. Fluorescent foci quantitation was conducted as described above.

## Results

### Design of split MutT

*E. coli* MutT is the product of the mutator *mutT* gene and catalyzes the hydrolysis of the mutagenic nucleotide 8-oxo-dGTP *in vitro* (11). The *in vivo* role of MutT is indicated by the mutator phenotype of *mutT* strains and the higher mutation frequency induced by exogenous 8-oxo-dGTP in *mutT* cells (13,38). The split MutT protein was designed based on its tertiary structure (30,31). First, we conducted linker mutagenesis experiments to confirm that MutT is divisible between residues 95 and 96. The two residues are located in a loop and a Gly-Ser ((GGGGS)_6_) linker was inserted between these amino acids (Supplementary Figure S1A). We purified the linker-inserted mutant protein with a His-tag by affinity chromatography and compared the hydrolyzing activities for 8-oxo-dGTP of the wild-type (wt) and mutant MutT proteins. The activity of the mutant protein was comparable to that of the wt enzyme (Supplementary Figure S1B). When 10 μM 8-oxo-dGTP was incubated with 1.5 nM MutT at 30°C for 10 min, the hydrolysis efficiencies were 10.8 (±0.2) and 7.1 (±1.2) % for the wt and mutant proteins, respectively, indicating that the protein is divisible between residues 95 and 96 (the mean values ± standard errors of three independent experiments). Hereafter, split MutT protein refers to the two fragments split at this site.

The assembly of the N-terminal (1–95) and C-terminal (96–129) fragments, abbreviated as Mu95 and 96tT, respectively, was visualized by the Fluoppi technology (Figure 1 and Supplementary Figure S2) (34). Mu95 and 96tT were fused to Ash (assembly helper tag)/hAG (Azami Green). When Ash and hAG associate through the interaction of Mu95 and 96tT, fluorescent foci are observed, as described in the Introduction section. We expected that the Mu95-96tT complex formation would be augmented by 8-oxo-dGTP. The 53rd codon in the gene encoding Mu95 was altered to GCC (Ala) to eliminate the hydrolysis function of MutT (31–33), and the codon usage was ‘humanized’ for both genes.

First, eight combinations of the fused proteins (Ash-Mu95/Mu95-Ash plus hAG-96tT/96tT-hAG or hAG-Mu95/Mu95-hAG plus Ash-96tT/96tT-Ash) were examined by transiently expressing the two proteins in human U2OS osteosarcoma cells. The nucleoside (8-oxo-dG) was added to the culture medium to increase the intracellular concentration of 8-oxo-Gua nucleotides (see the Discussion section). We hypothesized that an 8-oxo-Gua nucleotide promotes the association of Mu95 and 96tT, and hence that of hAG and Ash, resulting in more fluorescent foci when the combination(s) are functional. The cells were observed by fluorescent microscopy and the numbers of fluorescent foci were counted (Supplementary Figure S3). We judged that the combination of the *Ash-Mu95* and *hAG-96tT* genes gave a low background and an increase of foci after the 8-oxo-dG treatment.

### Increase in fluorescent foci upon oxidized dG and dGTP treatments

Next, the *Ash-Mu95* and *hAG-96tT* genes were integrated into the genomic DNA of U2OS cells (Figure 2A and B and Supplementary Figure S4). The *Ash-Mu95* and *hAG-96tT* genes are connected via the T2A linker gene, and a clone carrying the fused gene was selected (sMutT cells). The sMutT cells were treated with 8-oxo-dG and statistically significant foci formation (7.8 foci per cell cytoplasm) was observed (Supplementary Figure S5A, Figure 2C, and Supplementary Movie S1). The dG treatment did not affect the number of foci as compared to the untreated control (0.8 and 1.0 foci per cell cytoplasm, respectively). Although the mean values indicate that each cell has at least one focus, no foci were observed in almost all of the dG-treated and the untreated control cells as shown by the median values (Supplementary Figure S5A, Figure 2C). The foci were mainly distributed in the cytosol. In addition, 8-oxo-dGTP was introduced into cells by the osmotic pressure method (36). This method induced foci formation (∼40 foci per cell cytoplasm). However, foci were highly increased upon 8-oxo-dGTP treatment (83.0 foci per cell cytoplasm), indicating that the split MutT is a useful sensor for the oxidized nucleotide (Supplementary Figure S5B and Figure 2D). Again, the foci were mainly distributed in the cytosol.

**Figure 3.**
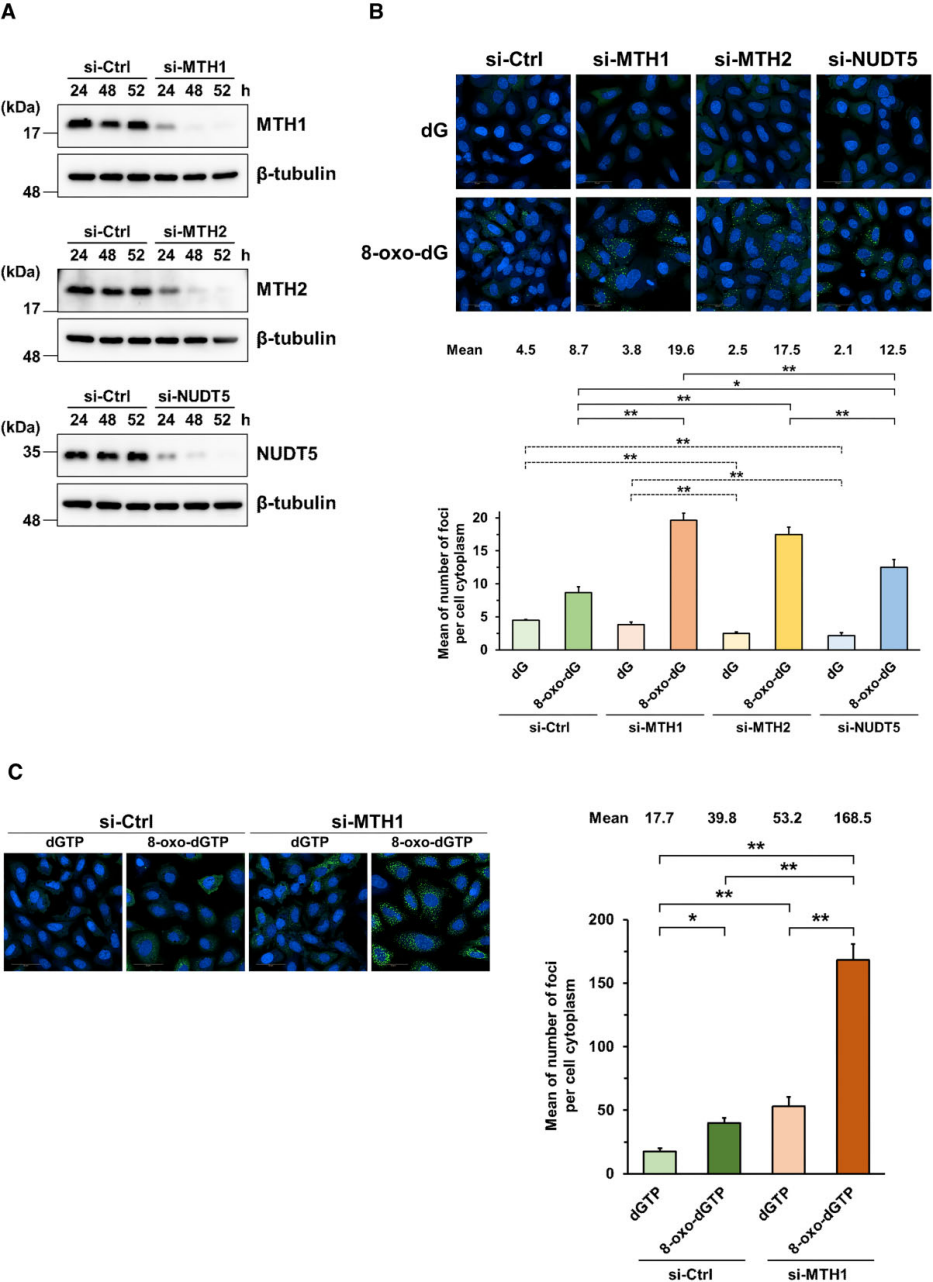
Increased fluorescent foci formation by knockdown of MTH1, MTH2, and NUDT5. (**A**) Knockdown of MTH1, MTH2, and NUDT5, confirmed by western blotting. (**B**) Increased fluorescent foci formation by 8-oxo-dG in MTH1-, MTH2-, and NUDT5-knockdown cells. (**C**) Increased fluorescent foci formation by 8-oxo-dGTP introduction in MTH1-knockdown cells. Stealth RNAi siRNA Negative Control, Med GC was used in the control experiments (si-Ctrl). The nuclei were stained by Hoechst 33342. The white bars in the images indicate 50 μm. Each mean values of the number of foci per cell cytoplasm obtained in three independent experiments shown in (B) Supplementary Figure S7A and (C) Supplementary Figure S7B were analyzed as single data. Data are represented as the means with standard deviations. *P< 0.05 and **P< 0.01 (Turkey–Kramer test). To avoid nonsense comparison, the experimental data of the dG and 8-oxo-dG groups were independently evaluated in (B).

Moreover, three human nucleotide pool sanitization enzymes, MTH1, MTH2 and NUDT5, which catalyze the hydrolysis of the 8-oxo-dG nucleotides, were separately knocked down by their respective siRNAs (Figure 3A and Supplementary Figure S6). The 8-oxo-dG treatment resulted in more foci formation than the control (dG treatment) and the knockdowns further increased the foci formation (8.7 foci per cell cytoplasm for the control group and 12.5–19.6 foci per cell cytoplasm for the knockdown groups, Supplementary Figure S7A, Figure 3B, and Supplementary Movie S2), supporting our interpretation that the foci were formed by intracellular 8-oxo-Gua nucleotides, the substrates for the knockdown targets. We next introduced 8-oxo-dGTP by the osmotic pressure method into the MTH1-knockdown and control cells (Supplementary Figure S7B and Figure 3C). As expected, foci formation was promoted in the knockdown cells. Thus, the established cells responded to the accumulation of intracellular 8-oxo-dGTP and formed fluorescent foci.

We manually counted the numbers of foci in living cells treated with 8-oxo-dG every five min in the time-lapse movies (Supplementary Figures S8 and Supplementary Figure S9). The foci increased from 5–10 min after 8-oxo-dG addition and reached the plateau after 20–30 min. The decrease of foci was suppressed by the knockdown of the nucleotide pool sanitization enzymes and the effect of MTH1 knockdown was most evident.

### Foci formation by oxidizing reagents and MTH1 inhibitors

**Figure 4.**
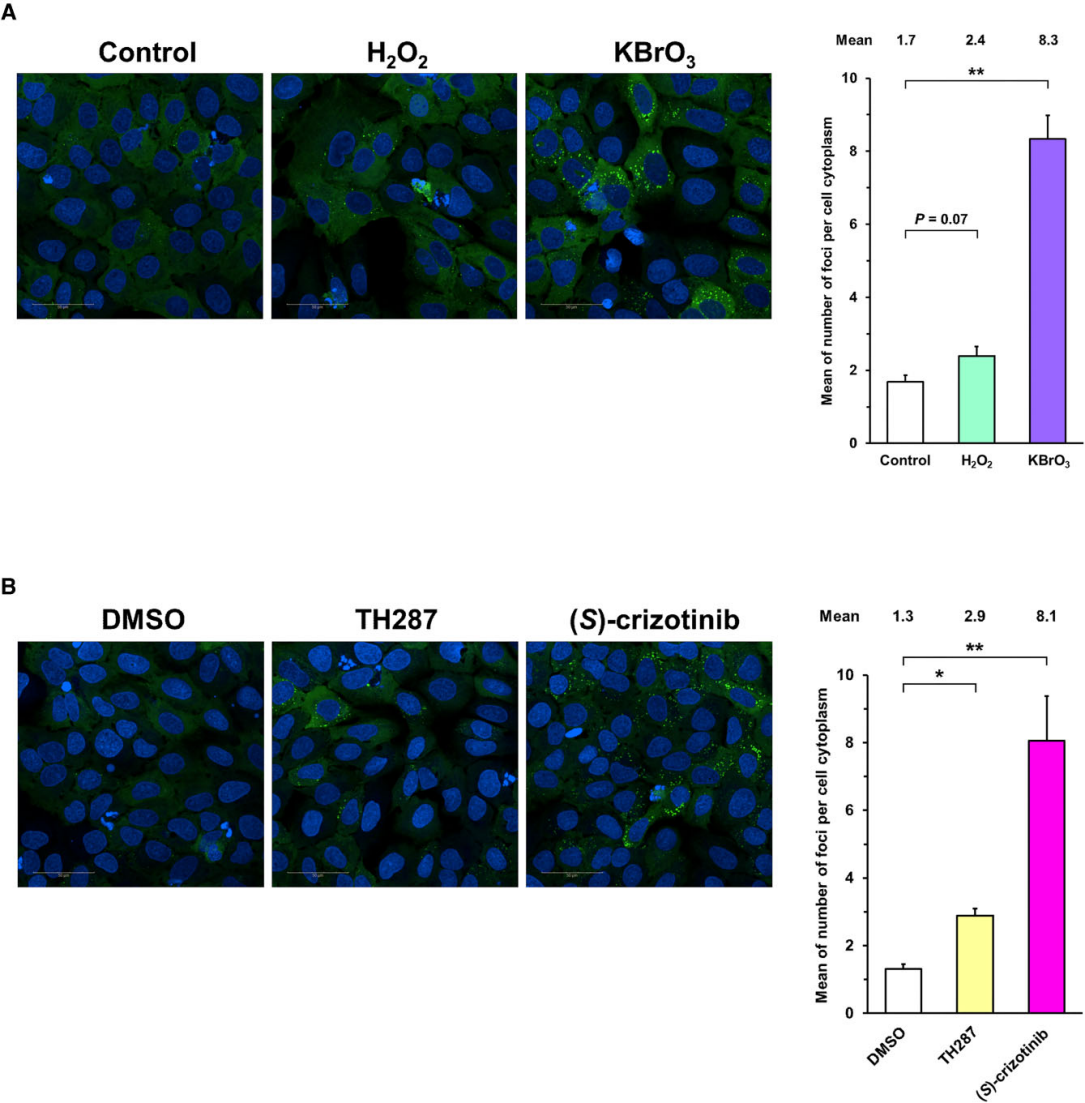
Fluorescent foci formation by oxidizing reagents and MTH1 inhibitors. (**A**) Treatments with H_2_O_2_ (30 μM) and KBrO_3_ (3 mM) for 6 h. (**B**) Treatments with MTH1 inhibitors, TH287 (100 μM) and (*S*)-crizotinib (5 μM) for 6 h. The nuclei were stained by Hoechst 33342. The white bars in the images indicate 50 μm. Each mean values of the number of foci per cell cytoplasm obtained in three independent experiments shown in (A) Supplementary Figure S10A and (B) Supplementary Figure S10B were analyzed as single data. Data are represented as the means with standard deviations. *P< 0.05 and **P< 0.01 (Dunnett test).

Next, the cells were treated with oxidizing reagents, H_2_O_2_ (30 μM) and KBrO_3_ (3 mM) (39,40). These compounds increased foci (Supplementary Figure S10A and Figure 4A), indicating that this system reflected intracellular 8-oxo-Gua nucleotide formation by oxidative stress and are useful as biomarkers. Note that the *P*-value between control and H_2_O_2_ is larger than 0.05 when the means obtained in three separate experiments are used as single data. However, the means and medians for the H_2_O_2_ groups in all experiments are larger than those for the control groups (Supplementary Figure S10A). Further increase in H_2_O_2_ concentration led to severe cell death.

Finally, the effects of known MTH1 inhibitors were examined. The sMutT cells were cultured in serum-free medium and treated with TH287 (100 μM) and (*S*)-crizotinib (5 μM) for 6 h (19,20). We observed foci formation upon treatments with these MTH1 inhibitors (Supplementary Figure S10B and Figure 4B). Therefore, fluorescent foci formation in cells expressing Ash-Mu95 and hAG-96tT would provide an excellent screening system for MTH1 inhibitors as anticancer therapeutics. Moreover, the system using living cells is favorable, since the activities of other enzymes such as MTH2 and NUDT5 are also reflected.

## Discussion

The system developed in this study uses the split MutT protein that was divided between residues 95 and 96 present in a loop region. One of the important features of the split MutT protein is that the association of the N-terminal (Mu95) and C-terminal (96tT) fragments is induced or highly promoted by the MutT substrate, 8-oxo-dGTP. As shown in Figure 2D, the 8-oxo-dGTP induction formed much more fluorescent foci than dGTP. Residues 23, 28, 35, 77, 78 and 119 of MutT participate in the binding to the 8-oxo-dG nucleotide (30,31). The former five amino acids and Asn-119 are located in the Mu95 and 96tT fragments, respectively. Asn-119 is involved in the recognition of O6 and N7-H of the 8-oxo-Gua base and seems highly important for the binding to the oxidized nucleotide. Thus, this residue might be a key to the assembly of the two fragments in the presence of 8-oxo-dGTP.

We observed the increased fluorescent foci by the 8-oxo-dG treatment of cells (Figure 2C). It should be noted that no phosphorylation of 8-oxo-dG in cell-free extracts of human leukemia cells was reported (41,42). However, Henderson and coworkers demonstrated that exogenous 8-oxo-dG labeled with ^14^C at the C8 position is metabolized in living human MCF-7 cells (43). The chromatographic behaviors of some compounds in the cell extract are similar to those of 8-oxo-rGTP and 8-oxo-dGTP and the radiocarbon is incorporated into the cellular RNA and DNA. Moreover, Immucillin H (a purine nucleoside phosphorylase inhibitor) and hydroxyurea (a ribonucleotide diphosphate reductase inhibitor) decrease the radiocarbon incorporation into RNA plus DNA (Immucillin H) and DNA (hydroxyurea). Thus, it seems that exogenous 8-oxo-dG is converted to the free base (8-oxo-Gua) by purine nucleoside phosphorylase and that 8-oxo-dGDP is produced from 8-oxo-rGDP by ribonucleoside diphosphate reductase. Hypoxanthine-guanine phosphoribosyltransferase and nucleoside diphosphate kinase possibly form 8-oxo-rGMP from 8-oxo-Gua and 8-oxo-rGTP/8-oxo-dGTP from the corresponding diphosphates, respectively, although no experimental evidence was shown in their study. The report of the phosphorylation of 8-oxo-rGDP/8-oxo-dGDP in a human Jurkat cell extract supports the latter reaction (41). Thus, the increase in fluorescent foci by 8-oxo-dG treatment of cells (Figure 2C) is probably induced by intracellular 8-oxo-rGTP/8-oxo-rGDP/8-oxo-dGTP/8-oxo-dGDP. However, Hayakawa *et al.* reported that mouse ribonucleoside diphosphate reductase did not convert 8-oxo-rGDP to 8-oxo-dGDP *in vitro* (27). This may be explained by the lack of cofactors or cellular integrity required for the reduction reaction of 8-oxo-rGDP (43) although we have no experimental evidence at this time. Further studies are necessary for the intracellular conversion from 8-oxo-dG to these nucleotides.

The ribo- and 2′-deoxyribo-nucleoside 5′-di-/tri-phosphates are substrates for MutT and these nucleotides bound to split MutT possibly induced foci formation. Previously, elevated transcriptional errors were observed in *mutT^–^ E. coli* cells (28). The expression of MTH1 or NUDT5 almost completely suppressed the errors. In contrast, no suppression was found upon MTH2 expression. These results indicate that MTH1 and NUDT5 but not MTH2 degrade the oxidized ribonucleotides. In this study, more fluorescent foci were formed in MTH2 knockdown cells upon the 8-oxo-dG treatment (Figure 3B). Thus, the increased foci in the MTH2 knockdown cells would reflect 8-oxo-dGTP accumulation in cells.

We observed the elevated foci upon the treatment of the sMutT cells with TH287 or (*S*)-crizotinib, low-molecular-weight MTH1 inhibitors (Figure 4B). The former was obtained by an *in vitro* MTH1 catalytic activity assay (19). The latter was screened by an *in vitro* thermal shift stability assay using SYPRO Orange (20). The high throughput screening using the cell lines like the sMutT cells possibly leads to the development of more potent inhibitors since the activities of MTH2 and NUDT5 are also reflected.

We observed ∼one focus when we treated sMutT cells with dG (Figure 2C). In contrast, the dGTP introduction induced ∼40 foci per cell cytoplasm (Figure 2D). This was due to the osmotic pressure method since the buffer without nucleotide also resulted in similar foci formation, suggesting oxidative stress.

We evaluated the number of foci in multiple sections of a cell, without considering the size. More quantitative evaluation would be possible when both the number and the three-dimensional size of foci are measured. Moreover, most of the foci were observed in the cytoplasm. This might be partially due to the cytosolic localization of either or both Ash-Mu95 and hAG-96tT that lack the nuclear localization signal.

It is unknown whether other split proteins that bind their substrates also exhibit properties similar to that of split MutT, the substrate-induced association of split fragments. However, this property could be used for visualization of the substrate of the protein of interest in living cells. For example, other split nucleotide-binding proteins as Orf135 and dUTPase could be used for the presence/quantitation of their substrates together with the substitution of catalytic amino acid residues (44,45).

In summary, we have demonstrated that the split MutT protein is a useful visualization system for oxidized dGTP and the related nucleotides. Fluorescent foci were abundant in cells after exogenous 8-oxo-dG and 8-oxo-dGTP treatment with/without MTH1 knockdown (Figures 2 and 3), and by treatments with oxidizing reagents (Figure 4A) and MTH1 inhibitors (Figure 4B). This is the first report on the visualization of damaged nucleotides in living cells and the system provides a biomarker for oxidative stress and a screening method for anticancer drugs.

## Supplementary Material

gkae371_Supplemental_Files

## Data Availability

Primers used for plasmid construction and siRNAs used in the knockdown experiments can be found in Supplementary Tables S1 and S3, respectively. All additional data are available from the authors upon request.
