# Supplementary material for: Visualization of oxidized guanine nucleotides accumulation in living cells with split MutT
Source: Nucleic Acids Res. 2024 May 13;52(11):6532–42. doi: 10.1093/nar/gkae371 (PMC11194108; doi:10.1093/nar/gkae371)
Supplement: gkae371_Supplemental_Files [file gkae371_supplemental_files.zip › Movie _legends_0311.docx]

**Movie S1**.

Live-cell imaging of sMutT cells with dG or 8-oxo-dG nucleoside treatment. The movie shows the responsiveness of sMutT cells after adding dG or 8-oxo-dG. The time in the movie indicates the elapsed time after the addition of dG/8-oxo-dG. The moment when the dG/8-oxo-dG was added is marked as 00 h: 00 m. The bars in the movies indicate 50 μm. The movies are related to Figure 2C.

**Movie S2**.

Live-cell imaging of MTH1-, MTH2-, and NUDT5-knockdown sMutT cells with dG or 8-oxo-dG nucleoside treatment. The movie shows the different responsiveness of each type of gene-knockdown sMutT cells after adding dG or 8-oxo-dG. The time in the movie indicates the elapsed time after the addition of dG/8-oxo-dG. The moment when the dG/8-oxo-dG was added is marked as 00 h: 00 m. The bars in the movies indicate 50 μm. The movies correspond to Figure 3B.
