# Supplementary material for: Visualization of oxidized guanine nucleotides accumulation in living cells with split MutT
Source: Nucleic Acids Res. 2024 May 13;52(11):6532–42. doi: 10.1093/nar/gkae371 (PMC11194108; doi:10.1093/nar/gkae371)
Supplement: gkae371_Supplemental_Files [file gkae371_supplemental_files.zip › Suppl_FigTables.pdf]

A

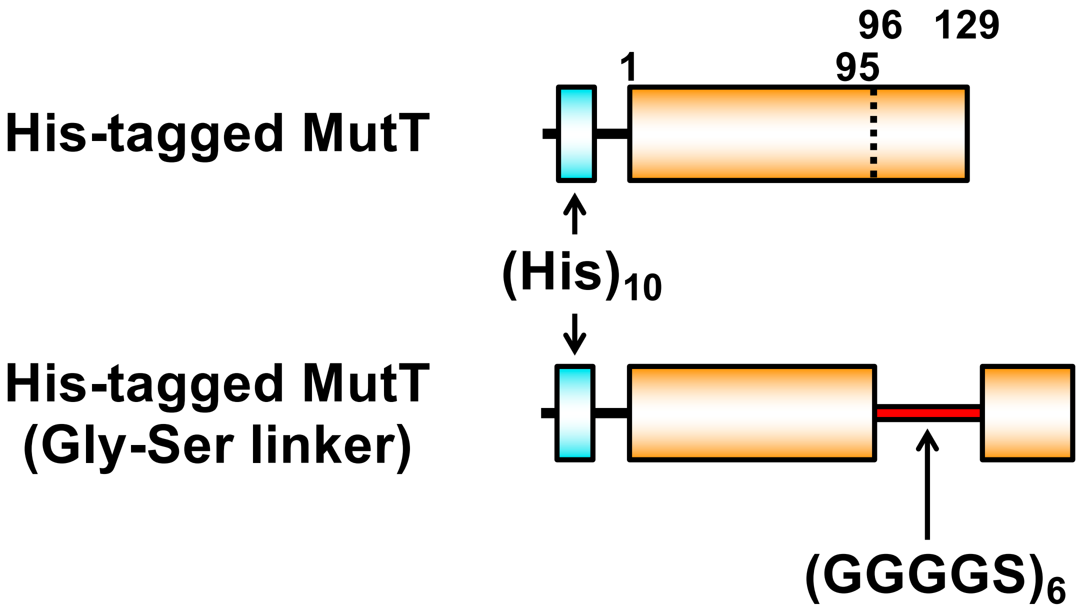
His-tagged MutT
96
129
1
95
(His)$_{10}$
His-tagged MutT
(Gly-Ser linker)
(GGGGS)$_{6}$

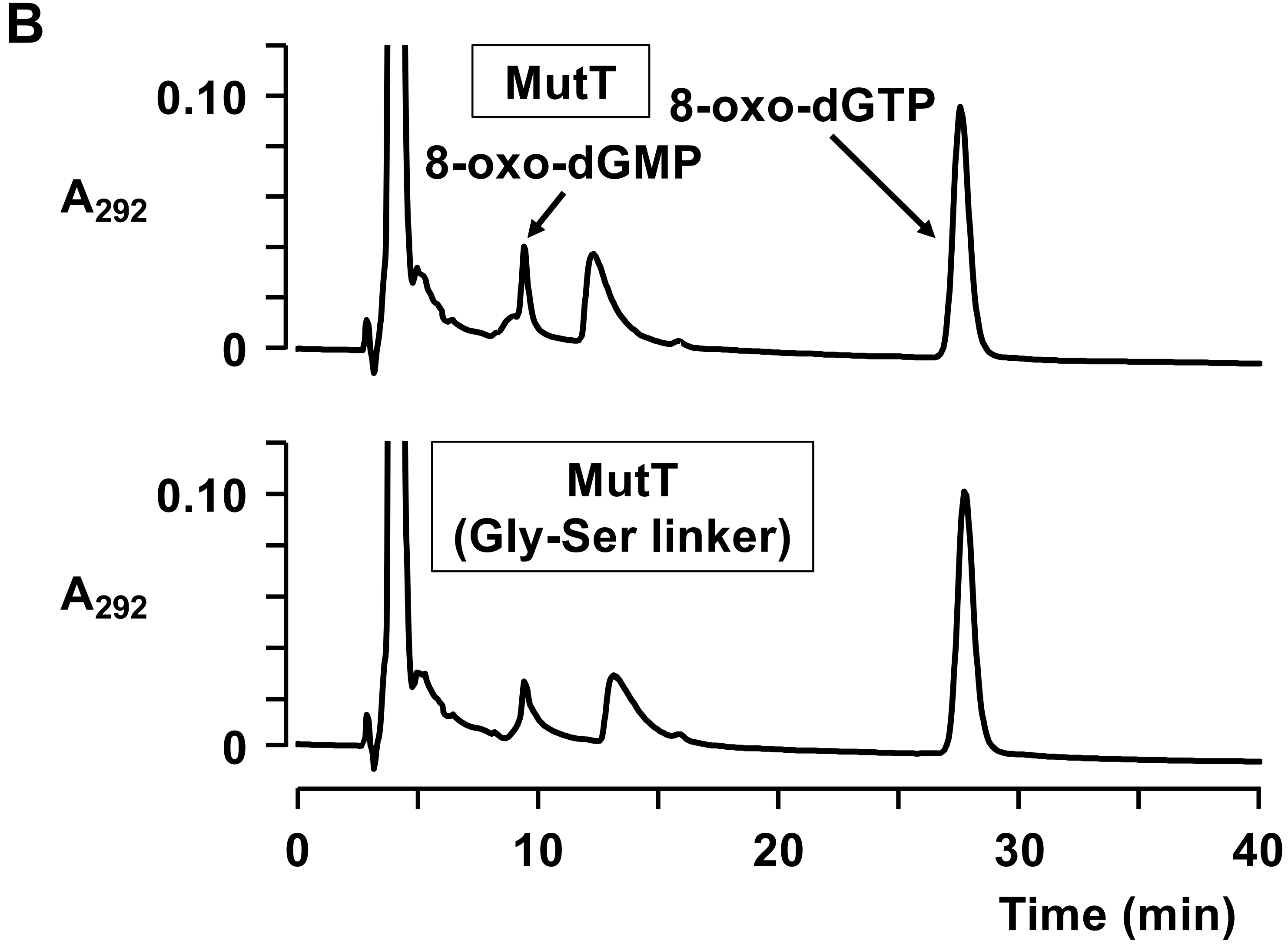
B
MutT
0.10
A292
0
8-oxo-dGMP
8-oxo-dGTP
MutT
(Gly-Ser linker)
0.10
A292
0
0
10
20
30
40
Time (min)

Supplementary Figure S1. Maintained 8-oxo-dGTPase activity of MutT upon Gly-Ser linker insertion between residues 95 and 96. (**A**) Structure of the wild-type and mutant MutT proteins. The $(His)_{10}$-tag was attached to the N-terminal of both proteins. The $(GGGGS)_6$-linker inserted between the 95th and 96th residues is shown as the red bar. (**B**) Hydrolysis of 8-oxo-dGTP by the MutT proteins, monitored by anion exchange HPLC. 8-Oxo-dGTP (10 μM) was incubated with 1.5 nM of MutT at 30°C for 10 min.

ATG	AAG	AAG	TTG	CAA	ATA	GCG	GTT	GGT	ATA
ATC	CGC	AAC	GAG	AAC	AAT	GAG	ATT	TTT	ATC
ACA	AGA	AGA	GCA	GCC	GAC	GCT	CAT	ATG	GCT
AAT	AAG	CTG	GAG	TTC	CCT	GGC	GGG	AAA	ATC
GAA	ATG	GGA	GAG	ACT	CCT	GAA	CAG	GCA	GTG
GTA	AGG	GCC	CTC	CAG	GAG	GAG	GTC	GGT	ATT
ACC	CCT	CAG	CAC	TTC	AGC	CTG	TTC	GAG	AAG
CTG	GAG	TAC	GAG	TTT	CCC	GAT	CGG	CAC	ATC
ACC	CTC	TGG	TTT	TGG	CTG	GTC	GAA	AGG	TGG
GAA	GGC	GAG	CCA	TGG	GGC	AAA	GAA	GGC	CAG
CCG	GGG	GAA	TGG	ATG	AGC	CTG	GTG	GGA	CTC
AAT	GCC	GAC	GAT	TTC	CCA	CCC	GCT	AAC	GAA
CCC	GTG	ATT	GCC	AAA	CTT	AAG	CGG	CTT	

Supplementary Figure S2. The *mutT* gene (Ala53) used in this study. Codons 1–95 and 96–129 are highlighted in yellow and green, respectively. Codon 53 is shown in red.

A

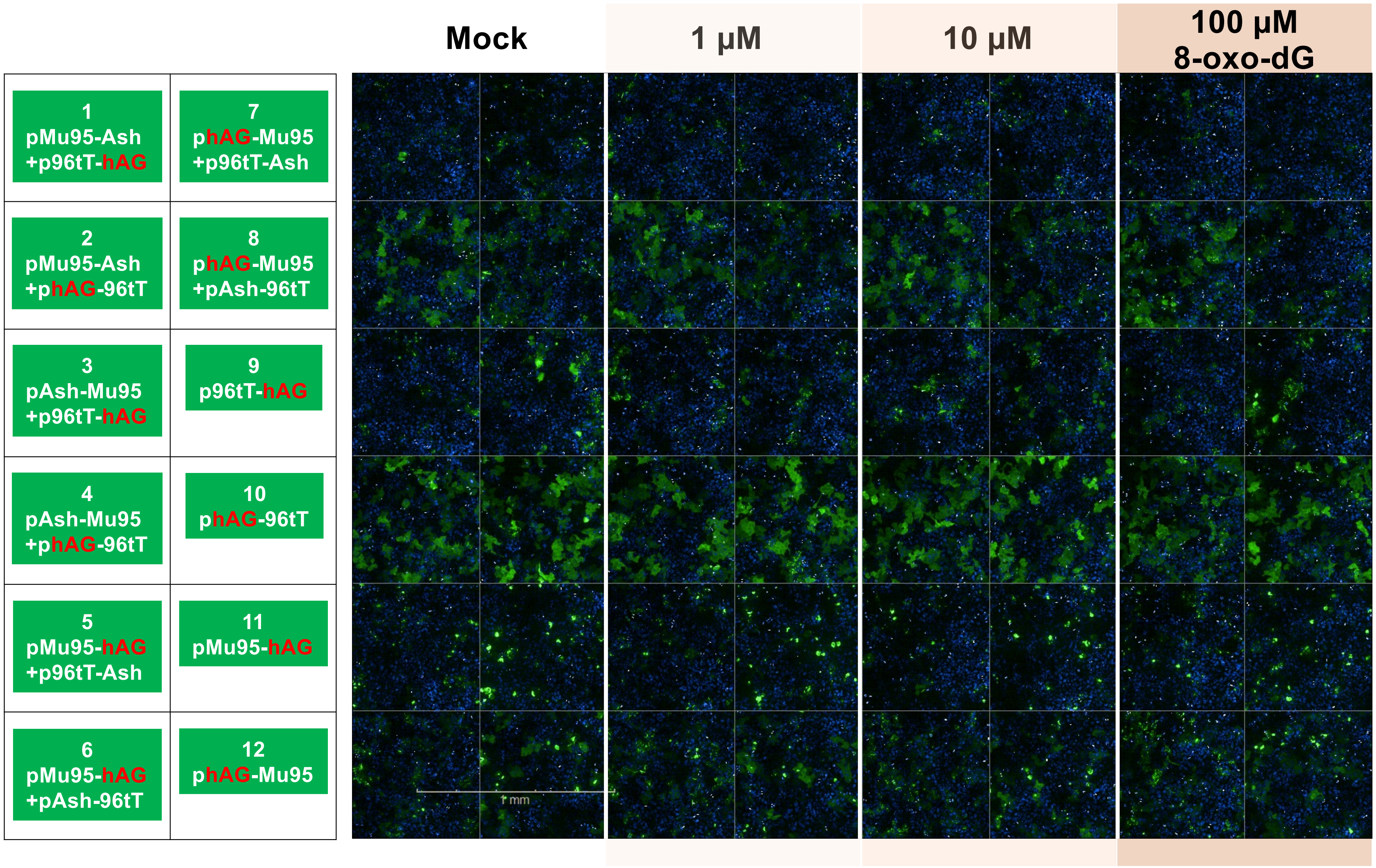
Mock
1 μM
10 μM
100 μM
8-oxo-dG
1
pMu95-Ash
+p96tT-hAG
7
phAG-Mu95
+p96tT-Ash
2
pMu95-Ash
+phAG-96tT
8
phAG-Mu95
+pAsh-96tT
3
pAsh-Mu95
+p96tT-hAG
9
p96tT-hAG
4
pAsh-Mu95
+phAG-96tT
10
phAG-96tT
5
pMu95-hAG
+p96tT-Ash
11
pMu95-hAG
6
pMu95-hAG
+pAsh-96tT
12
phAG-Mu95
1 mm

B

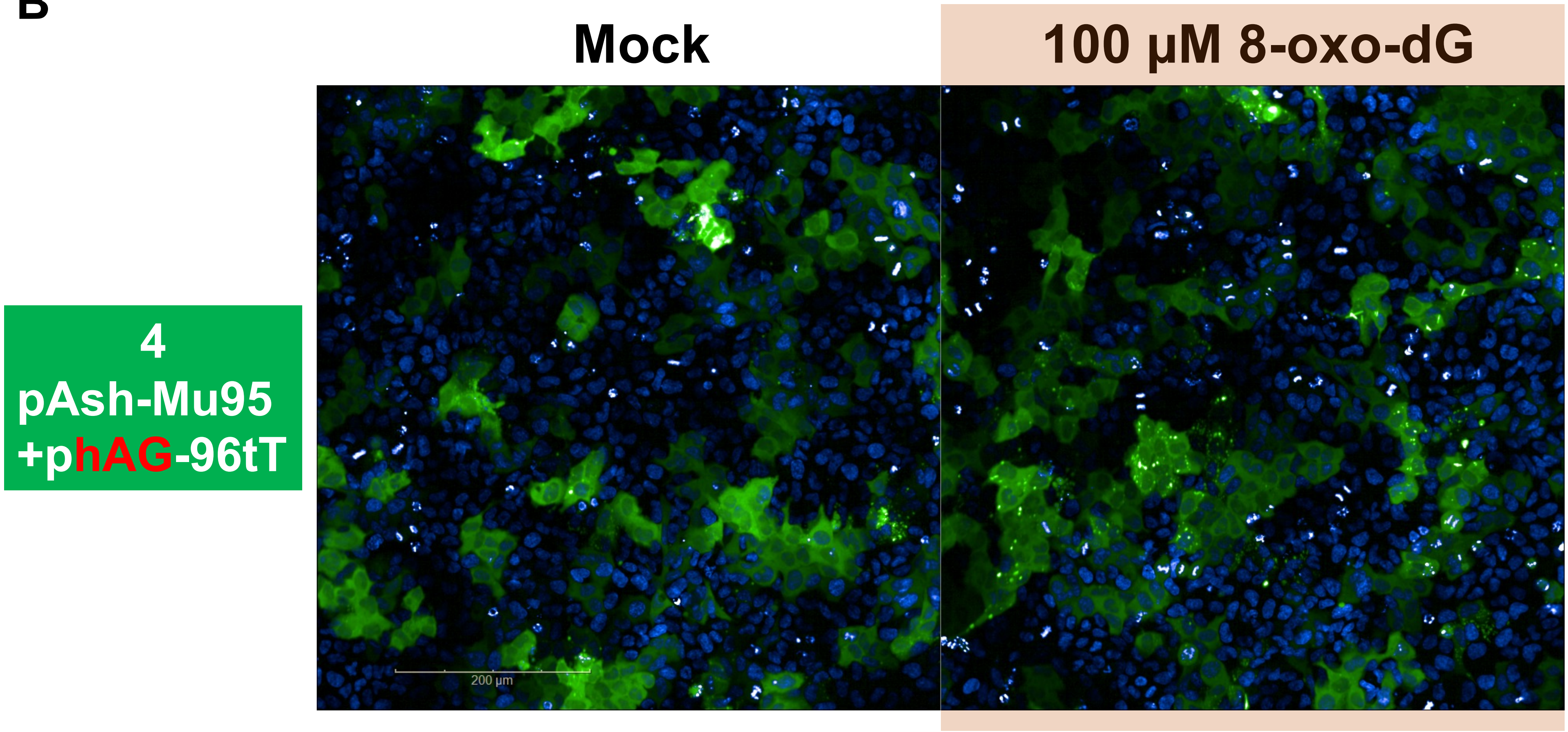

C

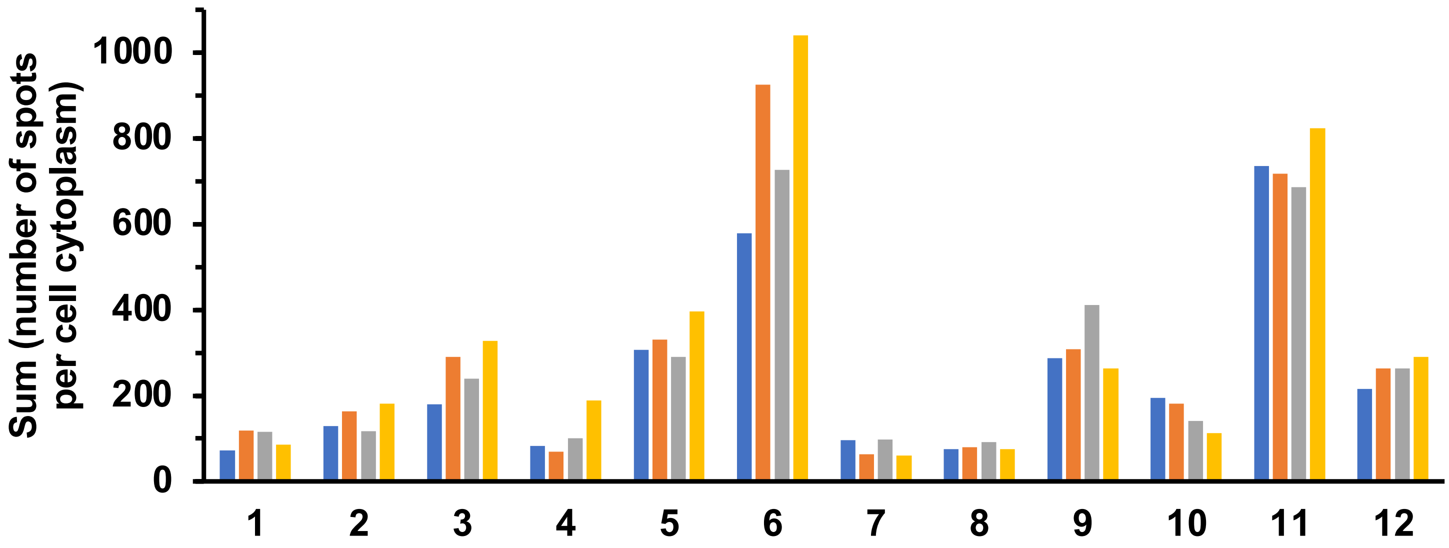

Sum (number of spots per cell cytoplasm)
1000
800
600
400
200
0
1
2
3
4
5
6
7
8
9
10
11
12

D

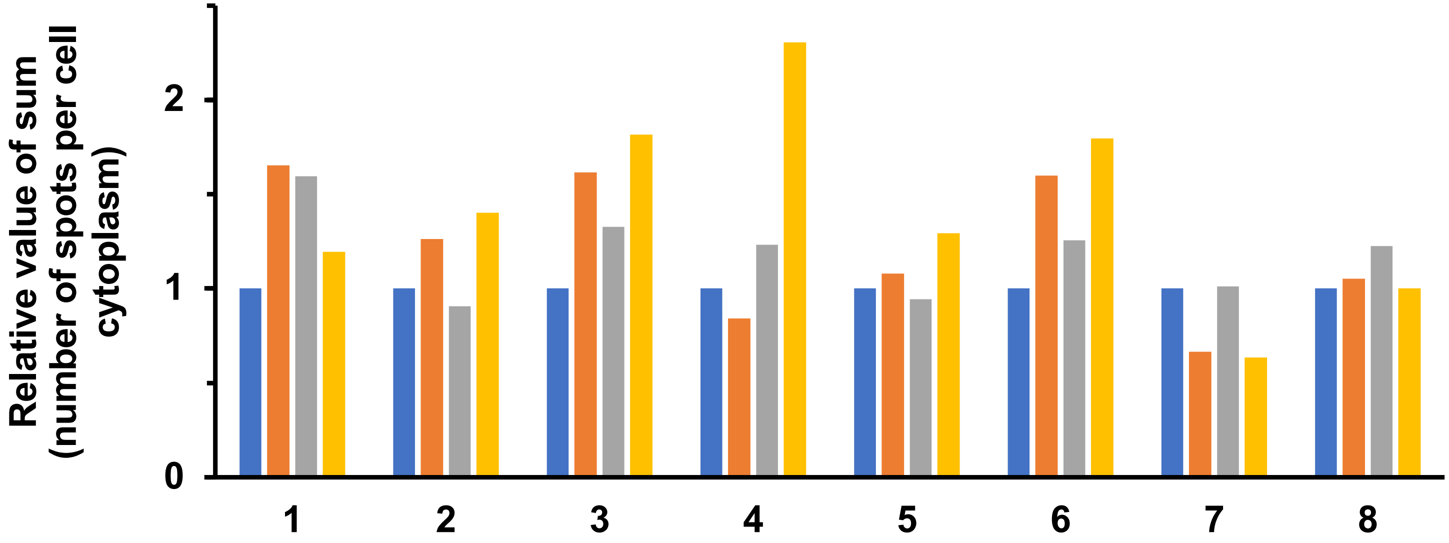

Relative value of sum (number of spots per cell cytoplasm)
2
1
0
1
2
3
4
5
6
7
8

Supplementary Figure S3. Screening for the optimal combination of Mu95/96tT and Ash/hAG by transient expressions in U2OS cells. The Mu95 and 96tT plasmids were transfected into U2OS cells by lipofection. The cells were treated with the 8-oxo-dG nucleoside. (**A**) Foci formation by 8-oxo-dG nucleoside treatment. (**B**) Representative images of foci formation by 100 μM 8-oxo-dG nucleoside treatment in cells transfected with pAsh-Mu95 and phAG-96tT plasmids. (**C, D**) Quantification of the number of foci among each combination of the plasmids. The nuclei were stained by Hoechst 33342. The white bars in the images indicate (**A**) 1 mm and (**B**) 200 μm. The combinations of plasmids are shown in the left side in (**A**). (**C**) Graph representing the comparison of the sum of the number of foci per cell cytoplasm among the combinations of plasmids. (**D**) Graph representing the relative value for each sum of the number of foci per cell cytoplasm, using mock as 1, among the combinations of plasmids. The numbers under the *x* axis correspond to the combinations of plasmids shown on the left side of (**A**). (**C** and **D**) Blue, mock; orange, 1 μM 8-oxo-dG; gray, 10 μM 8-oxo-dG; yellow, 100 μM 8-oxo-dG.

ATGGCGTCGCTCACCGTGAAGGCCTACCTTCTGGGCAAGGAGGACGCGGCGCGCGAGATTCGCCGCTTCAGCTTCTGTTGC
AGCCCCGAGCCTGAGGCGGAAGCCGAGGCTGCGGCGGGTCCGGGACCCTGCGAGCGGCTGCTGAGCCGGGTGGCCGCCCTG
TTCCCCGCGCTGCGGCCTGGCGGCTTCCAGGCGCACTACCGCGATGAGGACGGGGACTTGGTTGCCTTTTCCAGTGACGAG
GAATTGACAATGGCCATGTCCTACGTGAAGGATGACATCTTCCGAATCTACATTAAAGAGAAAACCGGTAATTCCGCTGAC
GGCGGCGGAGGATCGGGTGGTAGTGGTGGTTCAGGAGGAGGATCGACCCAAGGAGGATCCTCAGGTACCGGAACTGCAGGC
ATGAAGAAGTTGCAAATAGCGGTTGGTATAATCCGCAACGAGAACAATGAGATTTTTATCACAAGAAGAGCAGCCGACGCT
CATATGGCTAATAAGCTGGAGTTCCCTGGCGGAAAATCGAAATGGGAGAGACTCCTGAACAGGCAGTGGTAAGGGCCCTC
CAGGAGGAGGTCGGTATTACCCCTCAGCACTTCAGCCTGTTCGAGAAGCTGGAGTACGAGTTTCCCGATCGGCACATCACC
CTCTGGTTTTGGCTGGTCGAAAGGTGGGAAGGCGAGCCATGGGAGGGCAGAGGAAGTCTTCTAACATGCGGTGACGTGGAG
GAGAATCCCGGCCCTATGGTGAGCGTGATCAAGCCCGAGATGAAGATCAAGCTGTGCATGAGGGGCACCGTGAACGGCCAC
AACTTCGTGATCGAGGGCGAGGGCAAGGGCAACCCCTACGAGGGCACCCAGATCCTGGACCTGAACGTGACCGAGGGCGCC
CCCCTGCCCTTCGCCTACGACATCCTGACCACCGTGTTCCAGTACGGCAACAGGGCCTTCACCAAGTACCCCGCCGACATC
CAGGACTACTTCAAGCAGACCTTCCCCGAGGGCTACCACTGGGAGAGGAGCATGACCTACGAGGACCAGGGCATCTGCACC
GCCACCAGCAACATCAGCATGAGGGGCGACTGCTTCTTCTACGACATCAGGTTCGACGGCGTGAACTTCCCCCCCAACGGC
CCCGTGATGCAGAAGAAGACCCTGAAGTGGGAGCCCAGCACCGAGAAGATGTACGTGAGGGACGGCGTGCTGAAGGGCGAC
GTGAACATGGCCCTGCTGCTGGAGGGCGGCGGCCACTACAGGTGCGACTTCAAGACCACCTACAAGGCCAAGAAGGACGTG
AGGCTGCCCGACTACCACTTCGTGGACCACAGGATCGAGATCCTGAAGCACGACAAGGACTACAACAAGGTGAAGCTGTAC
GAGAACGCCGTGGCCAGGTACAGCATGCTGCCCAGCCAGGCCAAGACCGGTAATTCCGCTGACGGCGGCGGAGGATCGGGT
GGTAGTGGTGGTTCAGGAGGAGGATCGACCCAAGGAGGATCCTCAGGTACCGGAACTGCAGGCATGGGCAAAGAAGGCCAG
CCGGGGGAATGGATGAGCCTGGTGGGACTCAATGCCGACGATTTCCCACCCGCTAACGAACCCGTGATTGCCAAACTTAAG
CGGCTT

Supplementary Figure S4. The *Ash-Mu95-T2A-hAG-96tT* gene. The sequences of *Ash-Mu95*, *T2A*, and *hAG-96tT* are highlighted in blue, gray, and green, respectively.

A

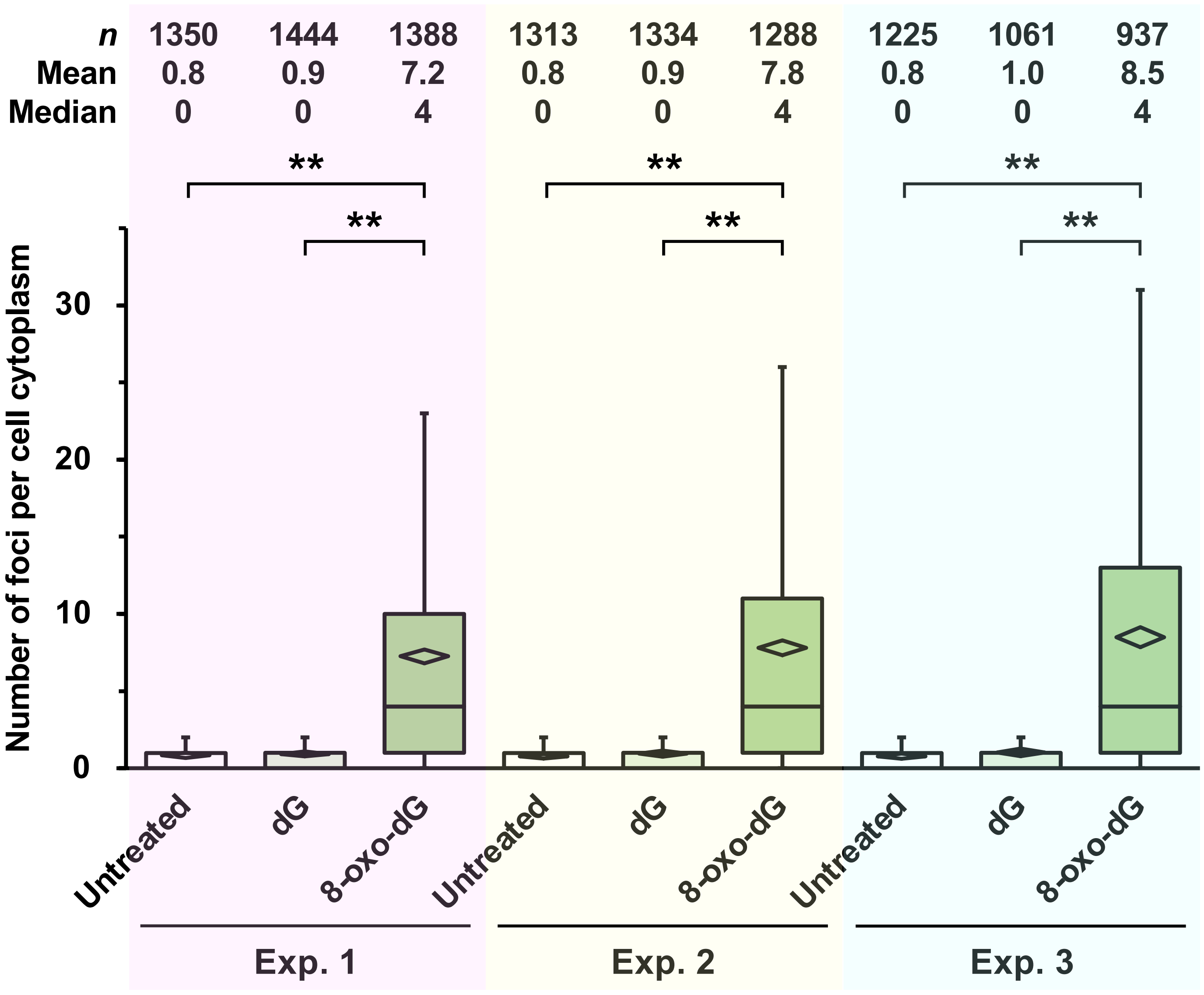
n 1350 1444 1388 1313 1334 1288 1225 1061 937
Mean 0.8 0.9 7.2 0.8 0.9 7.8 0.8 1.0 8.5
Median 0 0 4 0 0 4 0 0 4
**
Number of foci per cell cytoplasm
0
10
20
30
Untreated
dG
8-oxo-dG
Exp. 1
Exp. 2
Exp. 3

B

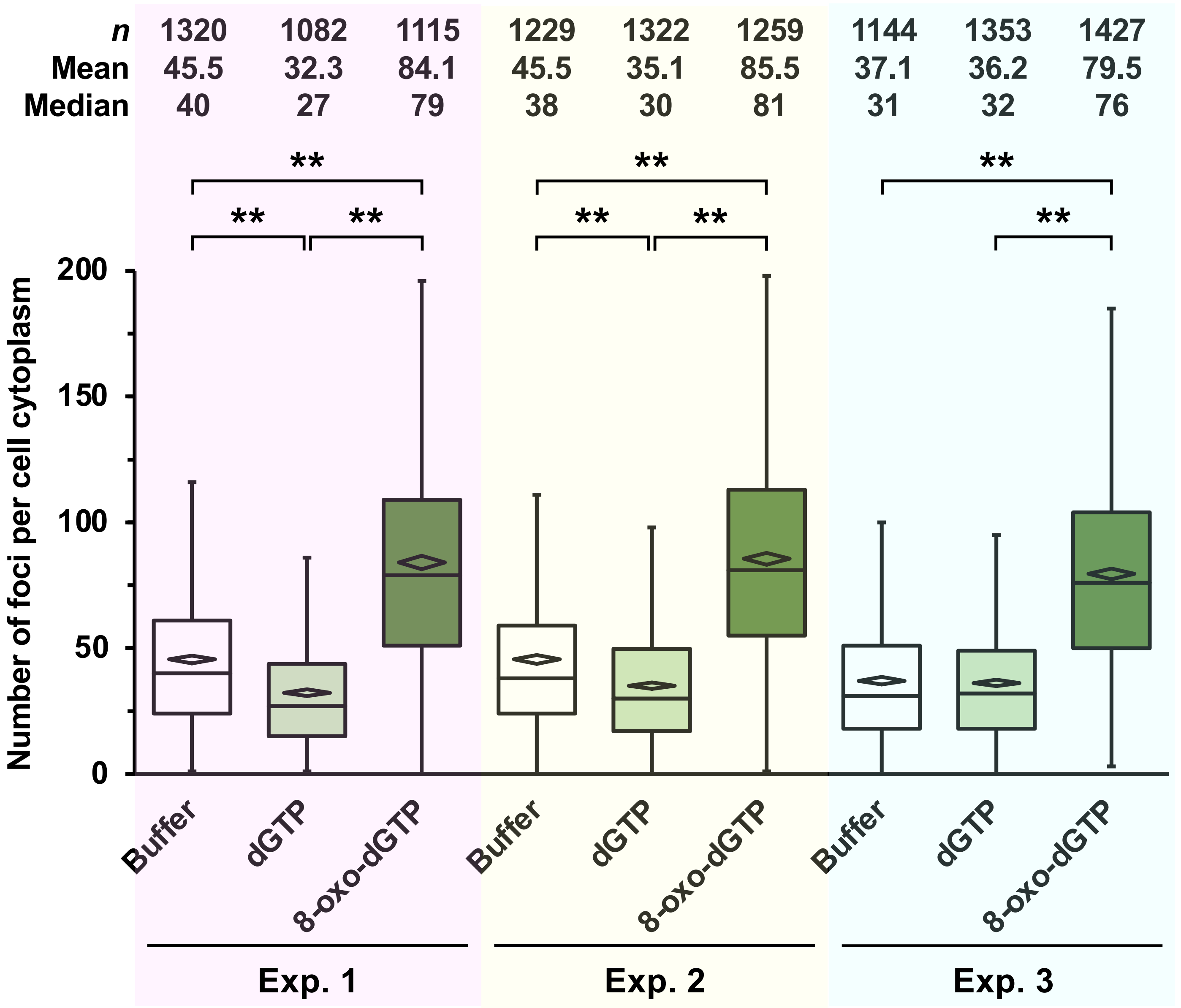

n 1320 1082 1115 1229 1322 1259 1144 1353 1427
Mean 45.5 32.3 84.1 45.5 35.1 85.5 37.1 36.2 79.5
Median 40 27 79 38 30 81 31 32 76
**
**
**
Number of foci per cell cytoplasm
200
150
100
50
0
Buffer
dGTP
8-oxo-dGTP
Exp. 1
Exp. 2
Exp. 3

Supplementary Figure S5. Fluorescent foci formation in sMutT cells expressing the split MutT. (**A**) Foci formation by 8-oxo-dG nucleoside treatment. (**B**) Foci formation by 8-oxo-dGTP introduction. Box-and-whisker plots represent the comparisons of the number of foci per cell cytoplasm between (**A**) dG and 8-oxo-dG or (**B**) dGTP and 8-oxo-dGTP. Diamonds and central lines in the boxplots represent the means and medians, respectively. The limits of the boxes correspond to the 0.25-0.75 quartiles, with whiskers extending to the maximum value of 1.5 times the interquartile range. ***P<0.01 (Steel-Dwass test).

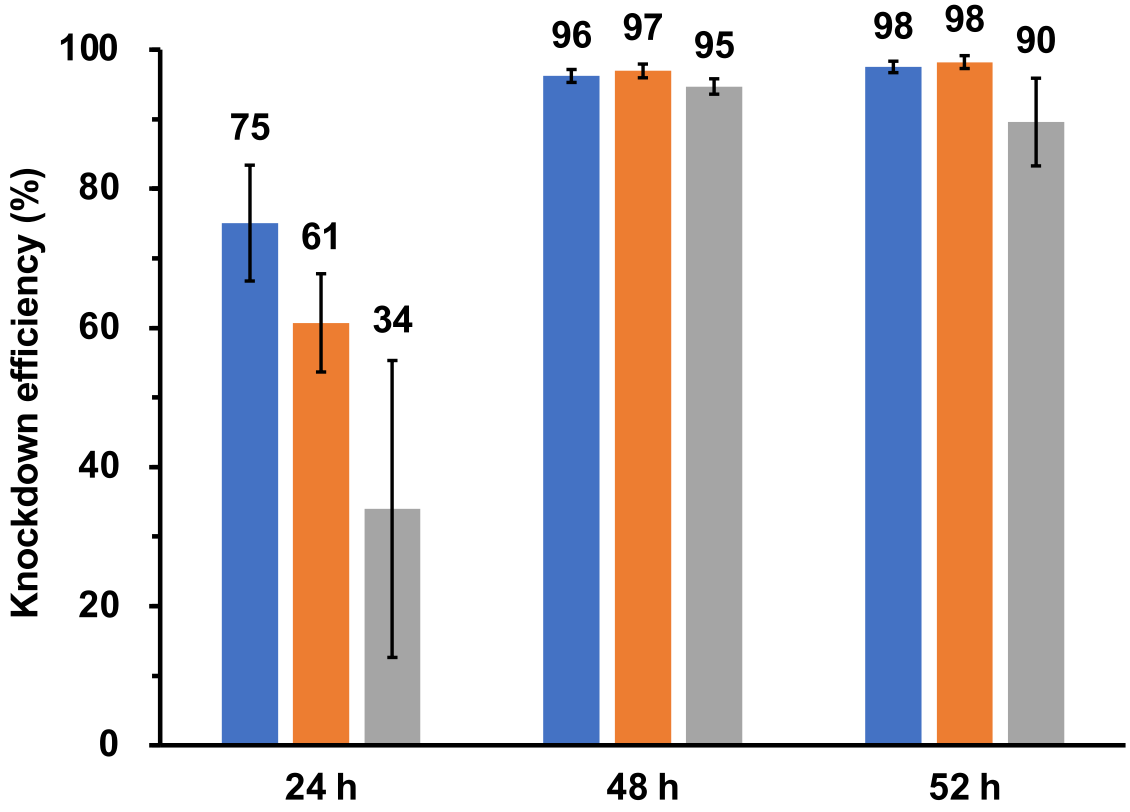

Supplementary Figure S6. Knockdown efficiencies of MTH1, MTH2, and NUDT5 by siRNAs. The levels of protein expression in sMutT cells at 24, 48, and 52 h after siRNA introduction were examined by western blot analysis and quantified with the ImageJ software (*37*). Data are expressed as the means ± standard errors. Blue, si-MTH1; orange, si-MTH2; gray, si-NUDT5.

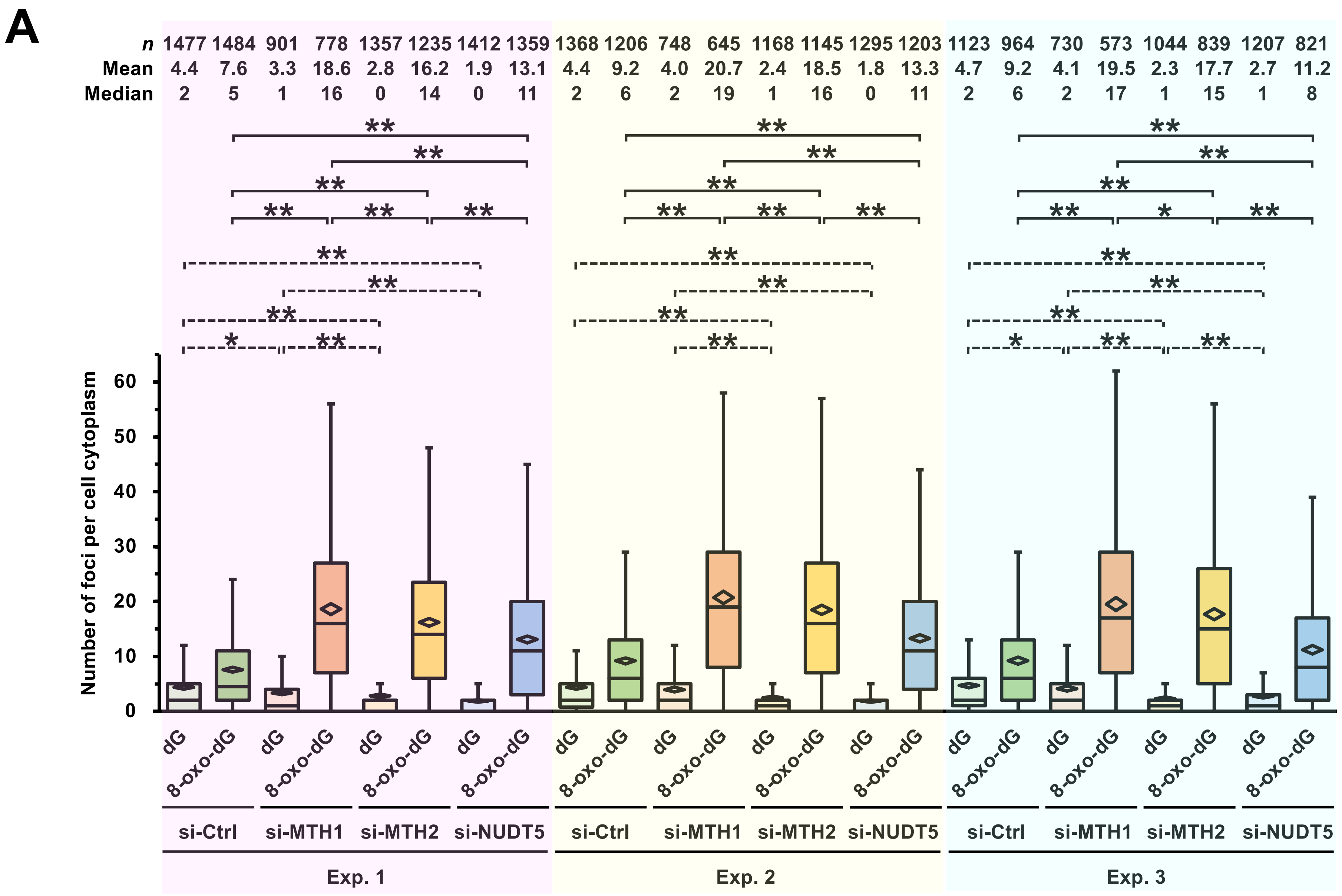

A
n 1477 1484 901 778 1357 1235 1412 1359 1368 1206 748 645 1168 1145 1295 1203 1123 964 730 573 1044 839 1207 821
Mean 4.4 7.6 3.3 18.6 2.8 16.2 1.9 13.1 4.4 9.2 4.0 20.7 2.4 18.5 1.8 13.3 4.7 9.2 4.1 19.5 2.3 17.7 2.7 11.2
Median 2 5 1 16 0 14 0 11 2 6 2 19 1 16 0 11 2 6 2 17 1 15 1 8
Number of foci per cell cytoplasm
0
10
20
30
40
50
60
dG
8-oxo-dG
si-Ctrl
si-MTH1
si-MTH2
si-NUDT5
Exp. 1
Exp. 2
Exp. 3

B

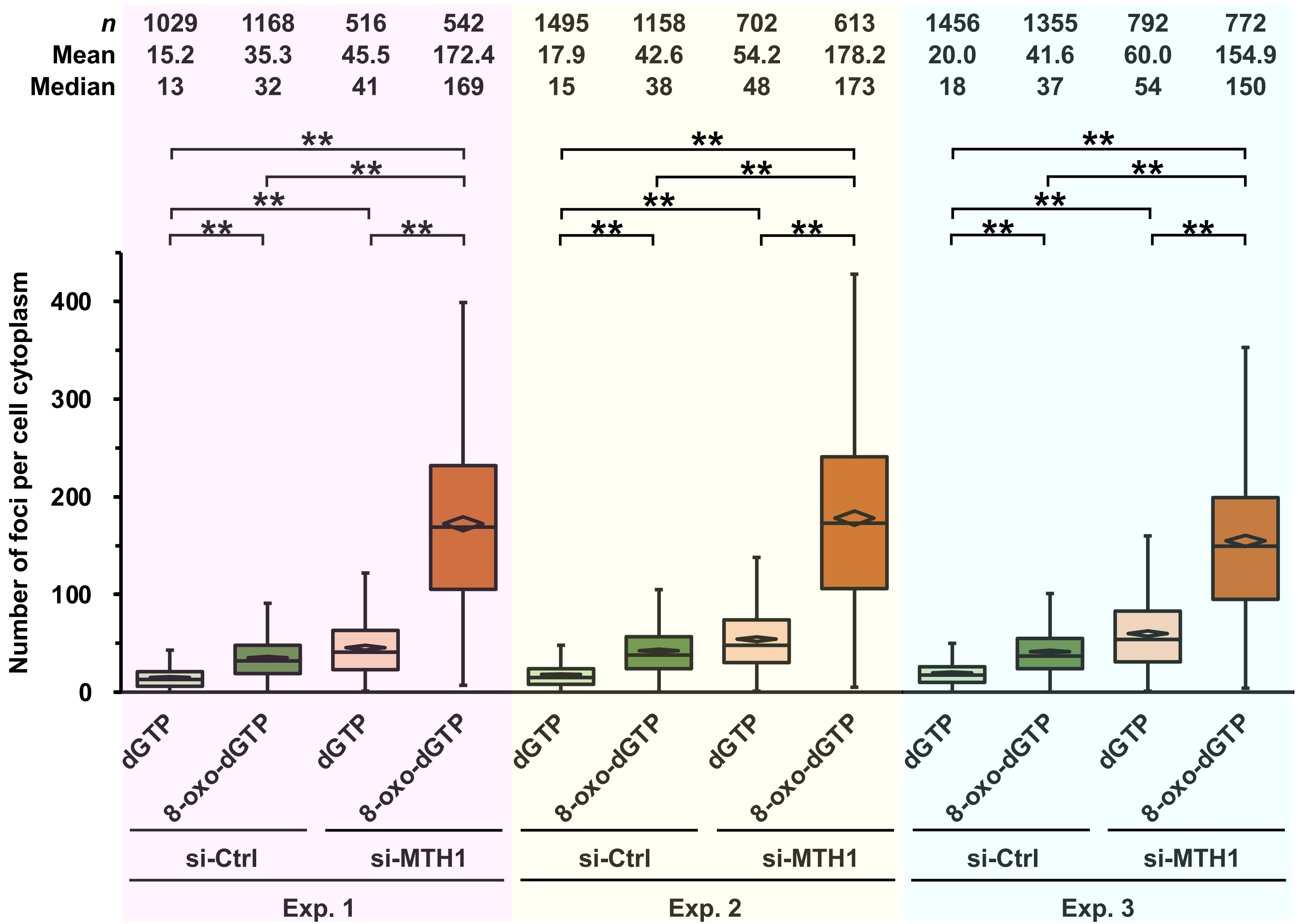
n
Mean
Median
1029 1168 516 542 1495 1158 702 613 1456 1355 792 772
15.2 35.3 45.5 172.4 17.9 42.6 54.2 178.2 20.0 41.6 60.0 154.9
13 32 41 169 15 38 48 173 18 37 54 150
**
Number of foci per cell cytoplasm
0
100
200
300
400
dGTP
8-oxo-dGTP
si-Ctrl
si-MTH1
Exp. 1
Exp. 2
Exp. 3

Supplementary Figure S7. Increased fluorescent foci formation by knockdown of MTH1, MTH2, and NUDT5. (**A**) Increased fluorescent foci formation by 8-oxo-dG in MTH1-, MTH2-, and NUDT5-knockdown cells. (**B**) Increased fluorescent foci formation by 8-oxo-dGTP introduction in MTH1-knockdown cells. Stealth RNAi siRNA Negative Control, Med GC was used in the control experiments (si-Ctrl). Box-and-whisker plots represent the comparisons of the number of foci per cell cytoplasm among (**A**) dG and 8-oxo-dG in MTH1-, MTH2-, and NUDT5-knockdown cells or (**B**) dGTP and 8-oxo-dGTP in MTH1-knockdown cells. Diamonds and central lines in the boxplots represent the means and medians, respectively. The limits of the boxes correspond to the 0.25-0.75 quartiles, with whiskers extending to the maximum value of 1.5 times the interquartile range. *$P<0.05$ and **$P<0.01$ (Steel-Dwass test). To avoid nonsense comparison, the experimental data of the dG and 8-oxo-dG groups were independently evaluated in (**A**).

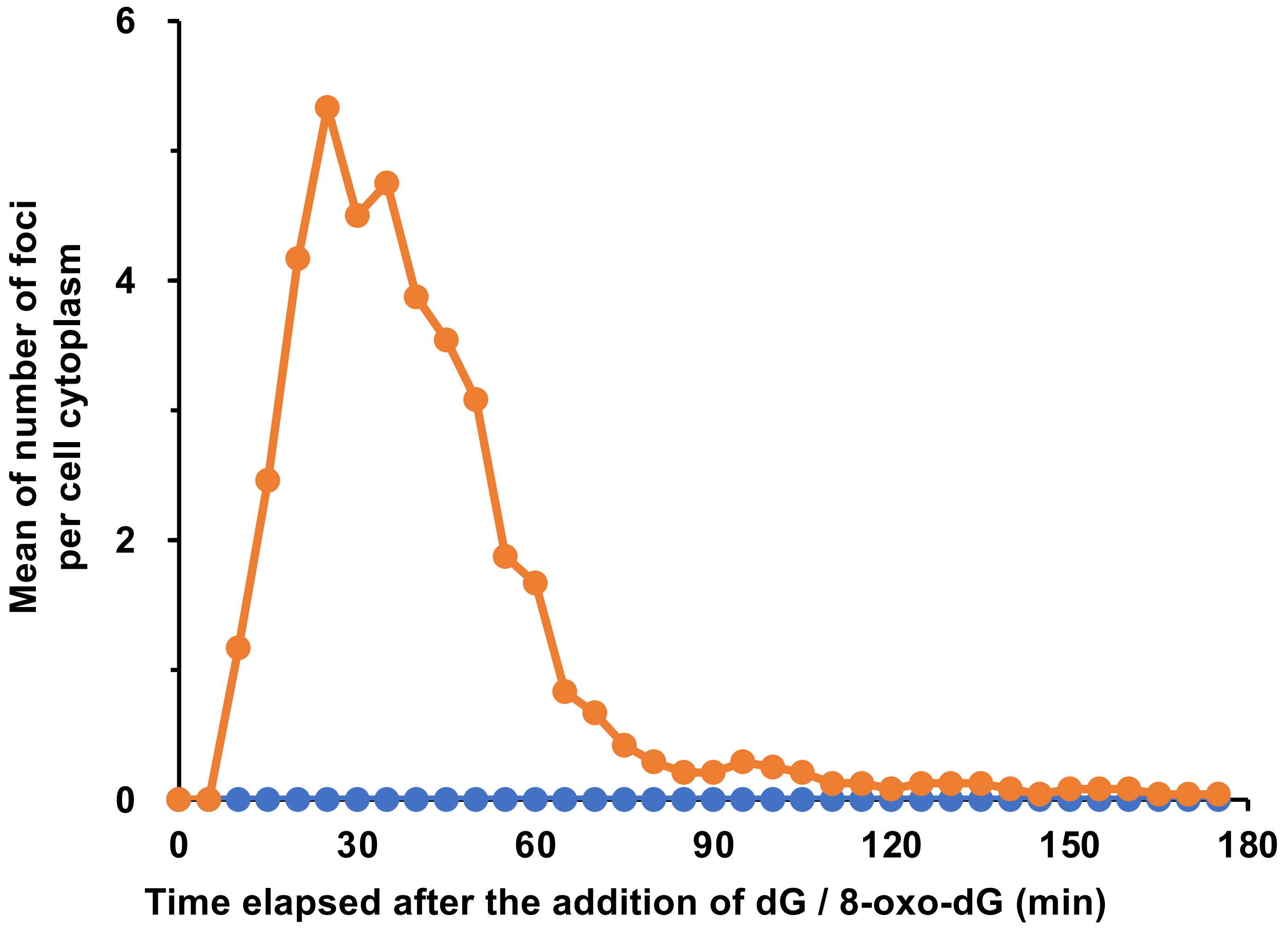

6
4
2
0
0
30
60
90
120
150
180
Mean of number of foci
per cell cytoplasm
Time elapsed after the addition of dG / 8-oxo-dG (min)

Supplementary Figure S8. Time course of the number of foci. The snapshots were extracted from Movie S1 and the foci in identical cells (~20 cells) were counted. Blue, dG treatment; orange, 8-oxo-dG treatment.

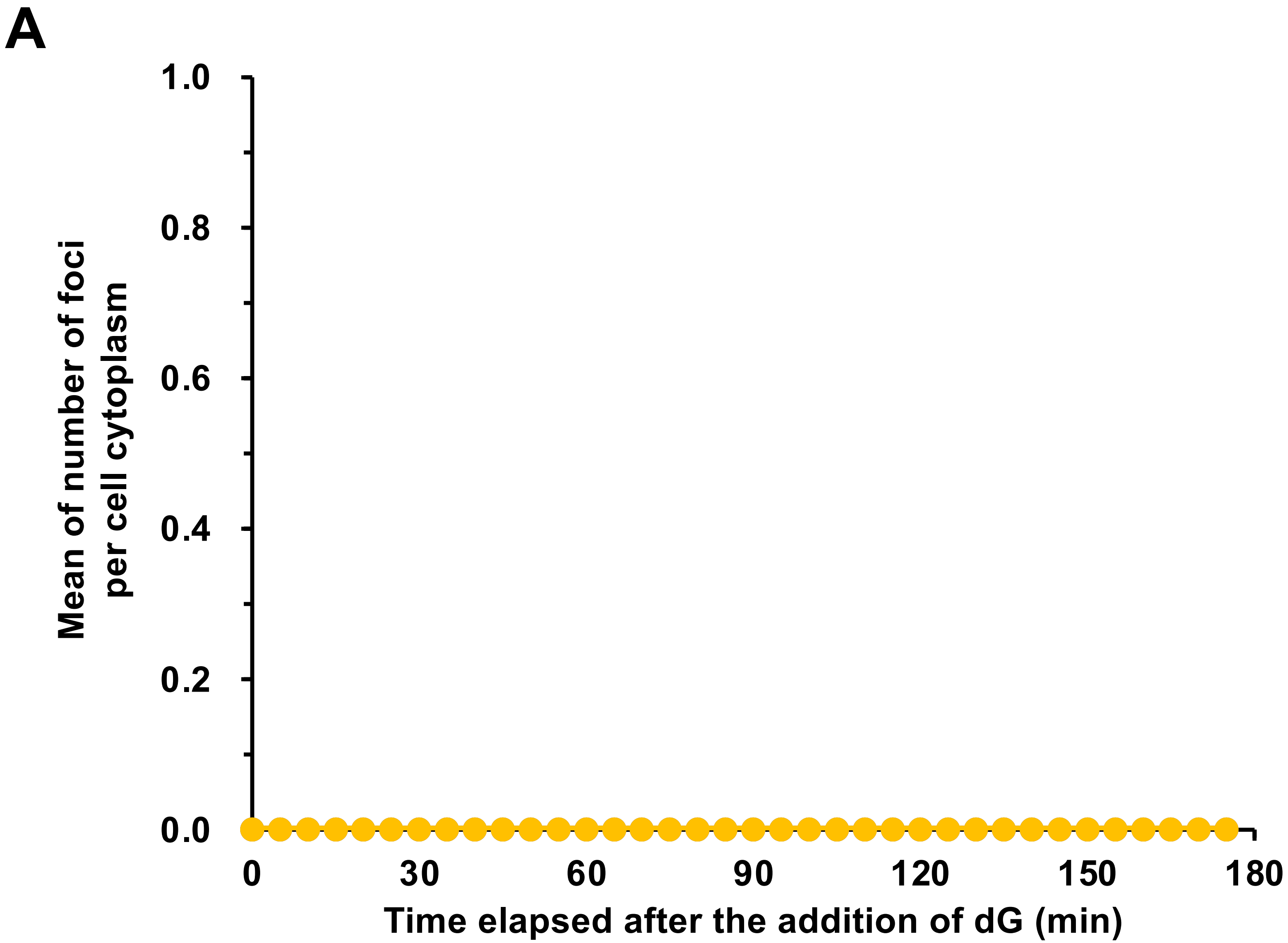
A
1.0
0.8
0.6
0.4
0.2
0.0
Mean of number of foci per cell cytoplasm
0
30
60
90
120
150
180
Time elapsed after the addition of dG (min)

B

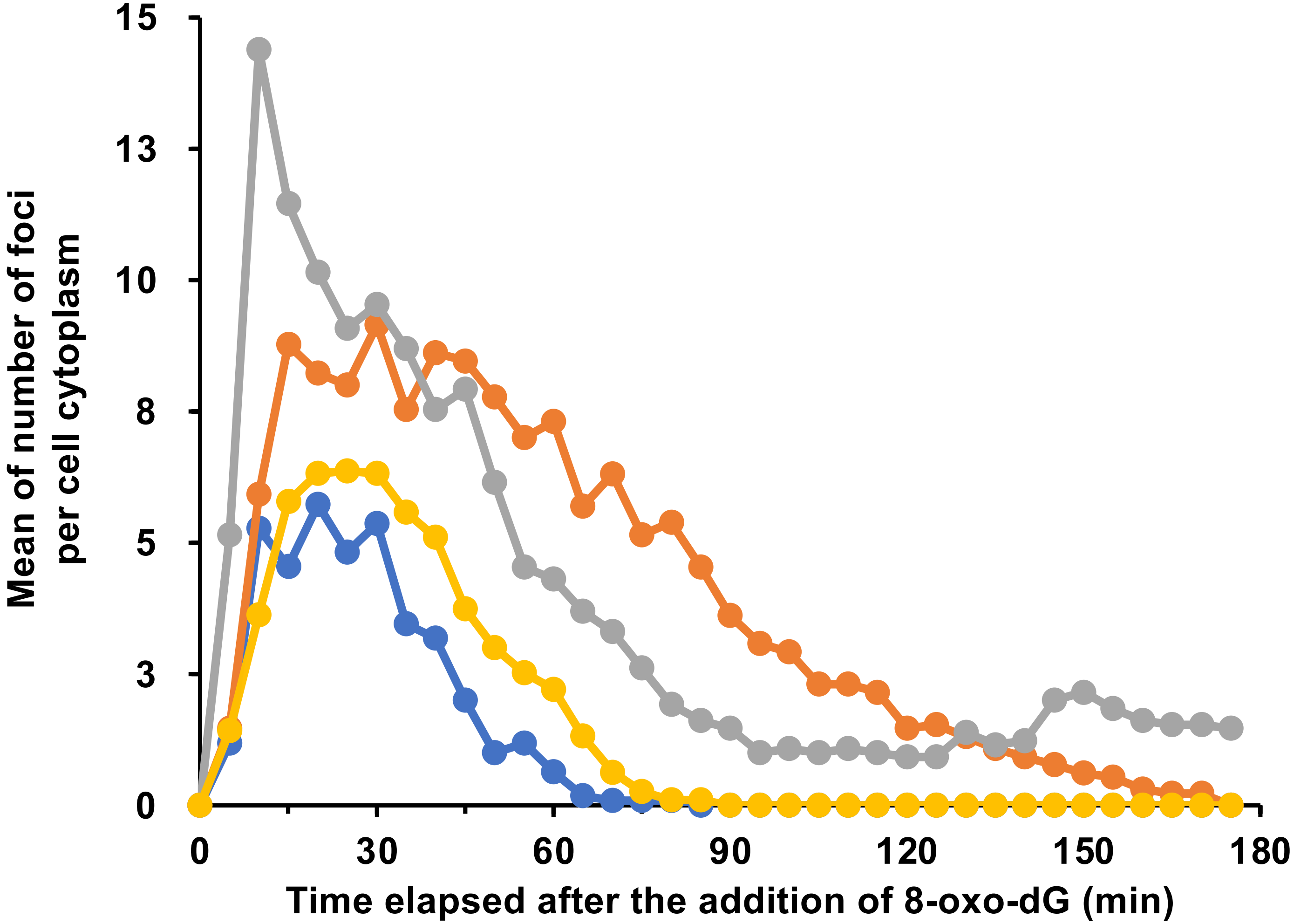
15
13
10
8
5
3
0
Mean of number of foci per cell cytoplasm
0
30
60
90
120
150
180
Time elapsed after the addition of 8-oxo-dG (min)

Supplementary Figure S9. Time course of the number of foci. The snapshots were extracted from Movie S2 and the foci in identical cells (~10 cells) were counted. (**A**) dG treatment and (**B**) 8-oxo-dG treatment. Blue, si-Ctrl (the control experiments); orange, si-MTH1; gray, si-MTH2; yellow, si-NUDT5.

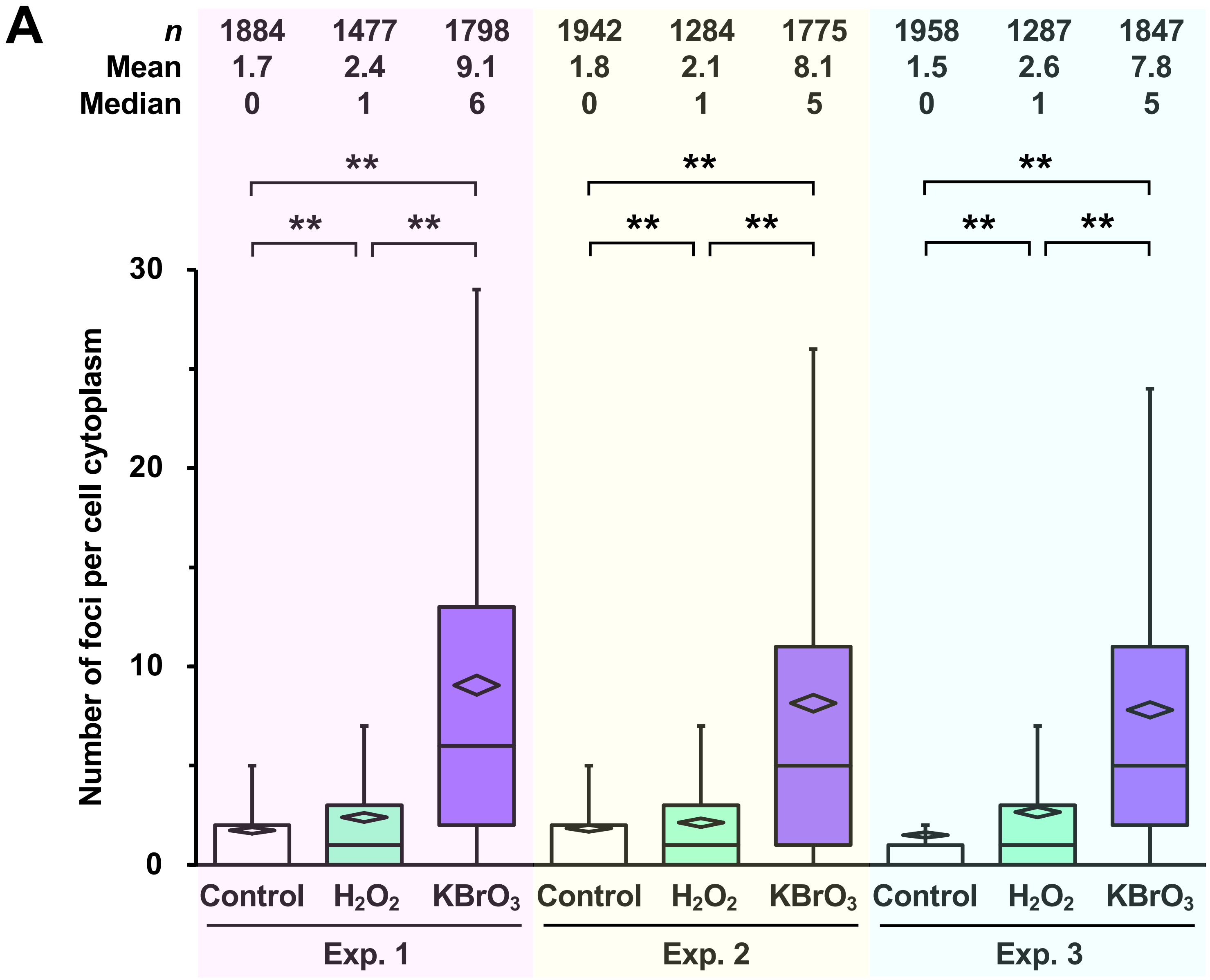
A
n
Mean
Median
1884
1477
1798
1942
1284
1775
1958
1287
1847
1.7
2.4
9.1
1.8
2.1
8.1
1.5
2.6
7.8
0
1
6
0
1
5
0
1
5
**
Number of foci per cell cytoplasm
30
20
10
0
Control
H_2O_2
$KBrO_3$
Exp. 1
Exp. 2
Exp. 3

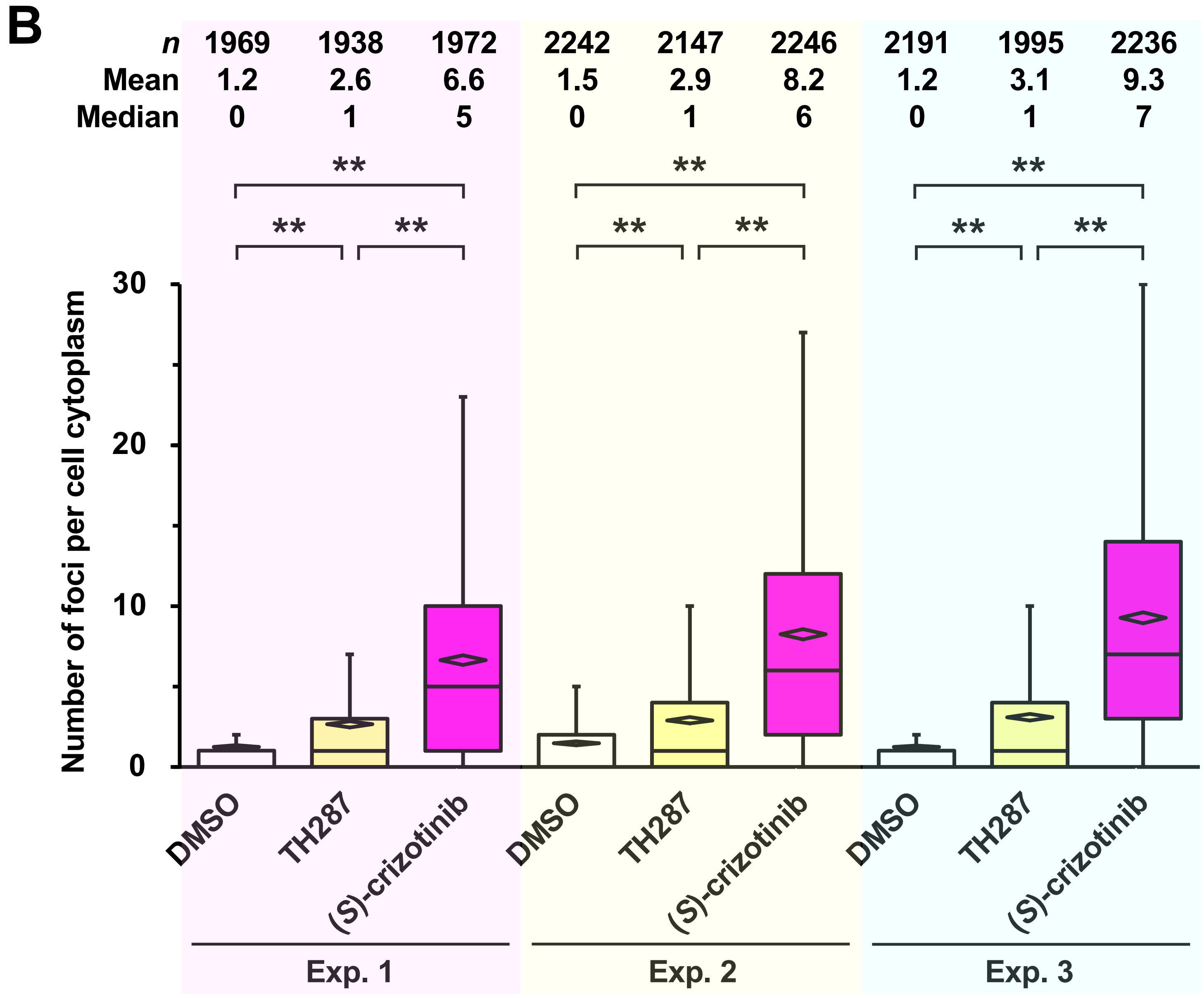

B
n 1969 1938 1972 2242 2147 2246 2191 1995 2236
Mean 1.2 2.6 6.6 1.5 2.9 8.2 1.2 3.1 9.3
Median 0 1 5 0 1 6 0 1 7
**
Number of foci per cell cytoplasm
30
20
10
0
DMSO
TH287
(S)-crizotinib
Exp. 1
Exp. 2
Exp. 3

Supplementary Figure S10. Fluorescent foci formation by oxidizing reagents and MTH1 inhibitors. (**A**) Treatments with H_2O_2 (30 μM) and $KBrO_3$ (3 mM) for 6 h. (**B**) Treatments with MTH1 inhibitors, TH287 (100 μM) and (*S*)-crizotinib (5 μM) for 6 h. Box-and-whisker plots represent the comparisons of the number of foci per cell cytoplasm among oxidizing reagents or (**B**) MTH1 inhibitors. Diamonds and central lines in the boxplots represent the means and medians, respectively. The limits of the boxes correspond to the 0.25-0.75 quartiles, with whiskers extending to the maximum value of 1.5 times the interquartile range. $^{**}P<0.01$ (Steel-Dwass test).

Supplementary Table S1 Primers used for plasmid construction.

Primer	Sequence (5' → 3')
For plasmids used in transient expression experiments	
Insertion of Mu95 into pAsh-MCL and phAG-MCL	
Mu95HC_Fwd	dAGGTACCGGAACTGCAGGCATGAAGAAGTTGCAAATAGCGG
C_Mu95HC_Rev	dGCTTATTCAAGCTTTGTTCTCGAGCTACCATGGCTCGCCTTCCCAC
Insertion of Mu95 into pAsh-MNL ver.2 and phAG-MNL	
Mu95HC_Fwd	dAGGTACCGGAACTGCAGGCATGAAGAAGTTGCAAATAGCGG
N_Mu95HC_Rev	dCTGATCCAAGCTTTGTTCTCGAGCCCCATGGCTCGCCTTCCCAC
Insertion of 96tT into pAsh-MCL and phAG-MCL	
96tT HC_Fwd	dAGGTACCGGAACTGCAGGCATGGGCAAAGAAGGCCAGCCGG
C_96tT HC_Rev	dGCTTATTCAAGCTTTGTTCTCGAGCTAAAGCCGCTTAAGTTTGGCAATC
Insertion of 96tT into pAsh-MNL ver.2 and phAG-MNL	
96tT HC_Fwd	dAGGTACCGGAACTGCAGGCATGGGCAAAGAAGGCCAGCCGG
N_96tT HC_Rev	dCTGATCCAAGCTTTGTTCTCGAGCCAAGCCGCTTAAGTTTGGCAATC
For pVOD_1 plasmid	
For amplification of CMV enhancer-promoter plus *Ash-Mu95HC*	
CMV-Ash- Mu95_Fwd	dGGAAAAGCTAGCCGTTACATAACTTACGGTAAATGGCCC
CMV-Ash- Mu95_Rev	dTTCCTCTGCCCTCCCATGGCTCGCCTTCCCA
For amplification of *T2A*	
Mu95-T2A- hAG_Fwd	dAGGCGAGCCATGGGAGGGCAGAGGAAGTCTTCTAACATG
Mu95-T2A- hAG_Rev	dTCACGCTCACCATAGGGCCGGGATTCTCCTC
For amplification of *hAG-96tTHC* plus poly(A) signal	
hAG-96tT- PA+6bp_Fwd	dGAATCCCGGCCCTATGGTGAGCGTGATCAAG
hAG-96tT- PA+6bp_Rev	dTCGCAGATCCTTACGCCTTAAGATACATTGATG
For amplification of *loxP*-insulator	
NsiI-MCS-loxP-Ins_Fwd	dTTACGGTGCTGCTATGCATAGCTAGCGGGCC
NsiI-MCS-loxP-Ins_Rev	dAAGTTATGTAACGGCTAGCTTTTCCCCGTATCC

Supplementary Table S1 Primers used for plasmid construction (*continued*).

Primer	Sequence (5' → 3')
For amplification of EF1α promoter-blasticidin S resistance gene-insulator-*loxP*	
EF1a-Bla-Ins-loxP-XhoI_Fwd	dTATCTTAAGGCGTAAGGATCTGCGATCGCTC
EF1a-Bla-Ins-loxP-XhoI_Rev	dCGGATCCGAATTCCTCGAGCCATGGAGATCTAC
For amplification of f1 *ori*-ampicillin resistance gene-pUC *ori*	
XhoI-f1ori-AmpR-Ori-ClaI_Fwd	dCCATGGCTCGAGGAATTCGGATCCGTCGAC
XhoI-f1ori-AmpR-Ori-ClaI_Rev	dCTAGCTATGCATAGCAGCACCGTAATCAGTAG

Supplementary Table S2 The constructed plasmid DNAs.

Plasmid	Encoded protein
For transient expression	
pAsh-Mu95HC	Ash-linker-Mu95
pMu95HC-Ash	Mu95-linker-Ash
phAG-Mu95HC	hAG-linker-Mu95
pMu95HC-hAG	Mu95-linker-hAG
pAsh-96tTHC	Ash-linker-96tT
p96tT HC-Ash	96tT-linker-Ash
phAG-96tTHC	hAG-linker-96tT
p96tT HC-hAG	96tT-linker-hAG
For stable expression	
pVOD_1	Ash-linker-Mu95-T2A-hAG-linker-96tT

Supplementary Table S3 siRNAs used in this study.

siRNA	Sequence (5' → 3')
si-MTH1	
sense	AUCUGAUCCAGCUGGAACCAGCAUG
antisense	CAUGCUGGUUCCAGCUGGAUCAGAU
si-MTH2	
sense	UUCCCAAGGAACCCACUCCCAACUU
antisense	AAGUUGGGAGUGGGUUCCUUGGGAA
si-NUDT5	
sense	AGAAGAUUCCGUUGGUUCUUGGCUC
antisense	GAGCCAAGAACCAACGGAAUCUUCU
